# Structure‐Aware Machine Learning for Polymers: A Hierarchical Graph Network for Predicting Properties From Statistical Ensembles

**DOI:** 10.1002/marc.202500671

**Published:** 2026-01-06

**Authors:** Julian Kimmig, Yannik Köster, Timo Koswig, Punith Raviswamy, Subhash V. S. Ganti, Stefan Zechel, Christopher Kuenneth, Ulrich S. Schubert

**Affiliations:** ^1^ Laboratory of Organic and Macromolecular Chemistry (IOMC) Friedrich Schiller University Jena Jena Germany; ^2^ Jena Center for Soft Matter (JCSM) Friedrich Schiller University Jena Jena Germany; ^3^ Linkdlab GmbH Jena Germany; ^4^ Faculty of Engineering Science University of Bayreuth Bayreuth Germany; ^5^ Bavarian Center For Battery Technology (BayBatt) University of Bayreuth Bayreuth Germany; ^6^ Helmholtz Institute for Polymers in Energy Applications Jena (HIPOLE Jena) Jena Germany; ^7^ Helmholtz‐Zentrum Berlin for Materials and Energy (HZB) Berlin Germany

**Keywords:** graph neural networks, kinetic Monte Carlo, machine learning, polymer informatics

## Abstract

Machine learning applications in polymer science are often inefficient due to molecular representations that neglect the inherent hierarchical and statistical nature of macromolecules. This work introduces a structure‐aware graph convolutional network (GCN) framework that addresses this limitation by treating polymer samples as statistical ensembles. The approach utilizes a hierarchical graph representation where nodes correspond to monomer units and explicitly integrates molecular mass distribution (MMD) data to account for sample dispersity. A key innovation is an ensemble‐based training strategy using topologically realistic graphs generated on‐demand via an optimized kinetic Monte Carlo simulation. The model's efficacy was validated on a broad range of tasks. On synthetic data, it achieved more than 98% accuracy in classifying complex polymer architectures. When applied to a large experimental dataset, the model predicts glass transition temperatures (*T*_g_) with high accuracy (R^2^ = 0.89 ± 0.01). Crucially, a fine‐tuning experiment demonstrated that the model could successfully learn the physically / chemically grounded relationship between *T*_g_ and molar mass by integrating MMD information. This work establishes a robust and physically realistic paradigm for polymer informatics, enabling more accurate property predictions and paving the way for accelerated in silico material design.

## Graphical abstract

This work presents a structure‐aware graph convolutional network that models polymers as statistical ensembles to predict macroscopic properties. By combining topologically realistic graphs generated via kinetic Monte Carlo simulations with explicit molar mass distributions, the framework achieves high accuracy in classifying architectures and predicting glass transition temperatures as an example use case.

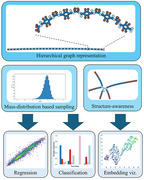

## 1 Introduction

The ongoing digital transformation is fundamentally reshaping materials research, driving toward an era where data‐driven methods supplement, and in some cases replace, traditional trial‐and‐error experimentation [1, 2, 3]. Within this paradigm shift, machine learning (ML) has emerged as a key technology for accelerating the design and discovery of new materials with tailored properties [4, 5, 6, 7]. Polymer science, characterized by its immense chemical and architectural design space, stands to benefit significantly from this evolution. However, the application of ML to polymers is fraught with challenges, including the scarcity of large, standardized datasets and the inherent complexity of macromolecular structures, which has meant that predictive modeling for polymers remains in its relative infancy [1, 8].

A central task in any “molecular” ML workflow is the translation of a chemical structure into a machine‐readable format. A foundational approach involves generating molecular fingerprints. These are fixed‐length vectors where each element typically signifies the presence or absence of a predefined chemical substructure or topological feature [9]. By systematically analyzing a molecule's structure, these algorithms produce a vector representation that is directly usable for many machine learning tasks, including polymer science [10].

Another, distinct data‐driven approach leverages language models that learn from sequential string representations. In this paradigm, transformer‐based architectures like polyBERT [11], PSMILES [12] or BigSMILES [13] treat polymer SMILES representations as a form of “chemical language.” By training on vast textual datasets, these models learn intricate structural and chemical patterns, enabling them to generate powerful representations for downstream tasks.

Alternatively, graph‐based methods have become state‐of‐the‐art for capturing more detailed topological information [14, 15, 16]. By representing a molecule as a graph–where atoms are nodes with feature vectors and bonds are edges–the full chemical topology is preserved. Graph convolutional networks (GCNs), designed to operate directly on graph data, have proven effective for capturing local as well as global information encoded in the chemical graph [14]. In a GCN, each node iteratively aggregates information from its neighbors in a process known as message passing, allowing the network to learn a rich representation of its local chemical environment [17]. These node‐level features are then pooled to generate a fixed‐size vector for the entire molecule, which is utilized for the property prediction [18, 19, 20].

However, the direct application of GCNs and conventional molecular representations developed for small molecules, that is, organic and inorganic substances, to polymers is inherently flawed due to the unique hierarchical nature of macromolecules. First, representing an entire polymer chain, which can contain millions of atoms, as a single graph is computationally prohibitive and inefficient [21]. Second, common featurization methods, such as extended‐connectivity fingerprints (ECFP), fail to capture critical polymer‐specific information [22]. While they can identify the presence of certain chemical motifs, they lose crucial context regarding monomer composition, mole fractions, end‐group identity, and, most importantly, the polymer's overall architecture and monomer sequence.

Compounding these representational challenges is a more fundamental distinction: Polymers, unlike small molecules with their fixed atomic counts, are intrinsically statistical systems. A given polymer sample is an ensemble of macromolecules possessing a distribution of chain lengths, compositions, and even topologies. Consequently, its macroscopic properties emerge from the collective behavior of this ensemble, not from a single, idealized structure alone [23]. Therefore, any representation that models a polymer as a single, monolithic graph is fundamentally incomplete. A truly structure‐aware model must also account for this dispersity. At a minimum, this requires incorporating statistical descriptors such as the number‐average (*M*_n_) and weight‐average (*M*_w_) molar masses, while a more complete approach would leverage information from the full molar mass distribution.

To overcome these significant limitations, a structure‐aware approach is required–one that is explicitly designed to understand and process the hierarchical and topological information unique to polymers. This study introduces a GCN‐based model developed specifically for predicting high‐level polymer characteristics from their graph representations, considering the statistical distribution of chain lengths, the topology and distribution of monomer units within the polymer molecule.

## 2 Methodology

### 2.1 Hierarchical Graph Representation

**FIGURE 1:**
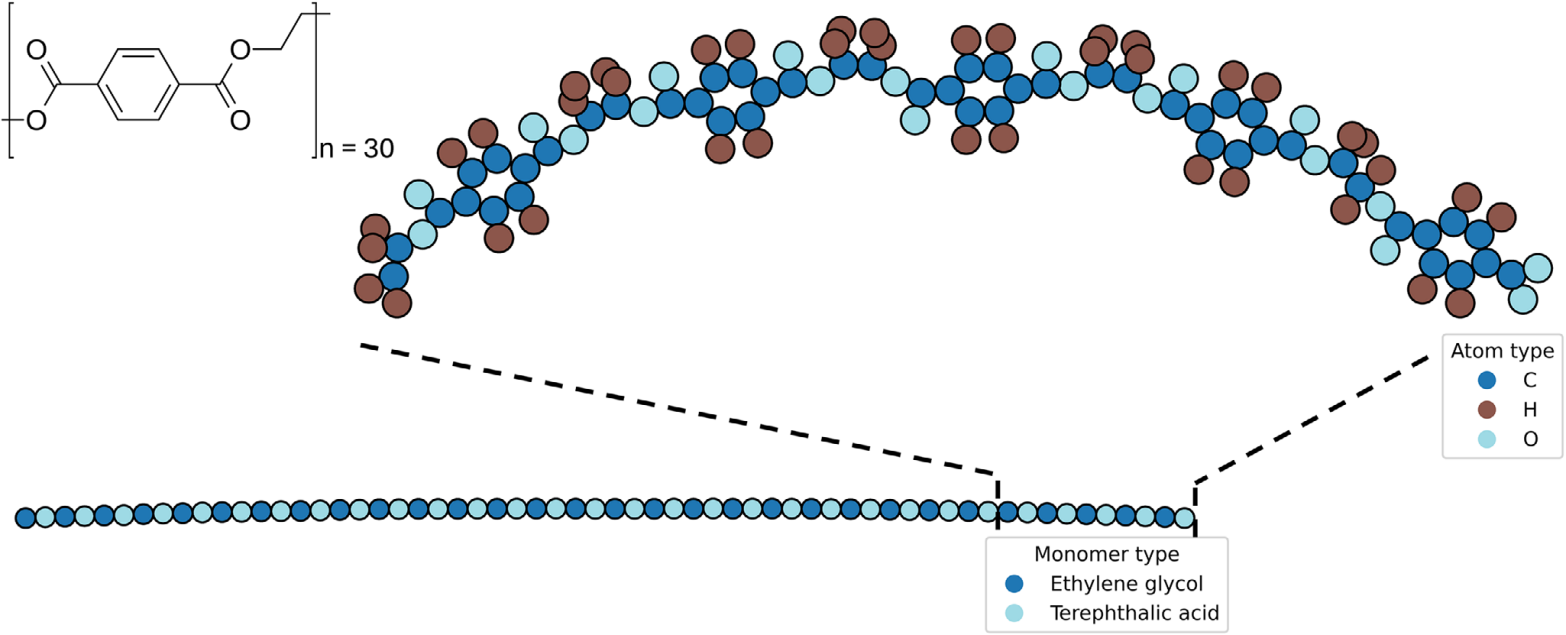
Repeating‐unit‐based vs. atom‐based graph representation. Visual example of the graph structures of poly(ethylene terephthalate) (top left: schematic representation of the structural formula) with 30 repeating units. While the repeating unit graph representation (bottom) requires only a few nodes to represent the graph an atom‐based graph representation (top right, truncated for visualization purposes) requires much more nodes to fully represent.

Unlike conventional graph‐based methods for small molecules that operate on an atomic level, our approach employs a hierarchical graph representation. In this scheme, each node in the graph denotes an entire repeating unit (i.e., a monomer), and the edges signify the covalent linkages between these units, thereby representing the polymer's chain connectivity (Figure 1). This coarse‐grained representation is computationally efficient and naturally aligns with the modular nature of polymers.

The feature vector for each node is designed to be flexible, allowing for the integration of various featurization techniques. These can range from traditional molecular fingerprints calculated for the monomer structure to more sophisticated, learned embeddings derived from chemical language models such as molBERT or the polymer‐specific systems like polyBERT [11, 24]. This allows the model to leverage rich, pre‐trained chemical knowledge at the monomer level.

For processing by a neural network, each polymer graph is formalized into a set of tensors. The nodes are represented by a node feature matrix *X* of the shape *N* × *F* where *N* is the number of monomers (nodes) in the polymer chain and *F* is the dimensionality of the feature vector for each monomer. The graph's topology is described by an edge index tensor, *E* of shape 2 × *M*, where *M* is the number of bonds (edges). As polymer graphs are undirected, each bond between monomers u and v is represented by two entries in the edge index, (*u, v*) and (*v, u*).

**FIGURE 2:**
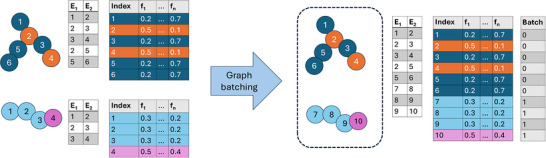
Schematic illustration of the graph batching process. Individual polymer graphs (left) are combined into a single super‐graph (right). Node features are concatenated vertically (for illustrative purposes colored with the respective node color), while edge indices are shifted by the cumulative node count of preceding graphs to prevent inter‐graph connections. A batch vector assigns each node to its original graph index for subsequent pooling operations.

For efficiency, multiple graphs should be processable by the network as a single batch, the issue is that the graph data is not equally shaped due to the high variance in the graphs. As a result, they cannot be simply stacked together like fixed size data formats (e.g., images, where all image tensors can be stacked to a single large one). Instead, a strategy is employed, where all individual graphs in a batch are combined into a single, larger disjoined graph (Figure 2). This is achieved by concatenating the node feature matrices of all graphs along the node dimension. For instance, a batch of k graphs would result in a single node feature tensor *X_batch_* of shape: $(\sum_{i=1}^{k}N_{i})\times F$.

Correspondingly, the edge index tensors are also concatenated. To maintain the integrity of each subgraph within the larger structure, the node indices within each graph's edge list are incremented by a cumulative sum of the number of nodes in the preceding graphs in the batch. This re‐indexing ensures that there are no spurious edges created between what were originally separate polymer graphs–effectively creating a block‐diagonal adjacency matrix. The final input to the GCN is, therefore, a single large graph object containing all nodes and edges from the batch, allowing for highly parallelized message passing operations across all graphs at once. To distinguish, which nodes belong to which original polymer, a batch assignment vector b is also constructed. This vector, of length $\sum_{i=1}^{k}N_{i}$, maps each node in the batched graph to the index of its original graph, enabling the final graph‐level pooling operations to be performed correctly for each individual polymer in the batch [25].

### 2.2 Integrating Molar Mass Distributions

A foundational concept that distinguishes polymer science from the study of small molecules is the intrinsic statistical nature of macromolecular chains in one polymer sample. Unlike a small molecule with a fixed, unambiguous molar mass, a polymer sample is a statistical ensemble. It comprises a collection of individual polymer chains possessing a distribution of chain lengths and, consequently, a distribution of molar masses. This molar mass distribution (MMD, described by the dispersity) is not a minor detail but a critical determinant of the macroscopic properties of a polymer. Key characteristics such as viscosity, tensile strength, toughness, and the glass transition temperature are profoundly influenced by the average size and shape of this distribution [23, 26, 27, 28, 29, 30, 31, 32]. Therefore, any predictive model that aims to be physically realistic must move beyond representing a polymer as a single, idealized structure and instead account for the collective properties of its underlying ensemble.

Experimentally, the MMD is mostly determined using size exclusion chromatography (SEC) [33]. This technique separates dissolved polymer molecules based on their hydrodynamic volume as they pass through a column packed with porous gel.

While SEC provides a relatively easy way to determine the MMD, this data is usually not available in large‐scale datasets or literature, where only the summary statistics (*M_n_* and *M_w_*) are reported.

To overcome this data scarcity and to ensure our model is trained on statistically representative ensembles, our framework generates high‐fidelity MMDs through simulation. This approach explicitly decouples the generation of the MMD from the generation of the graph topology. When full experimental MMD curves are not available, we leverage the reported *M_n_* and *M_w_* values to parameterize well‐established statistical distribution models, such as the Schulz–Zimm or, as is common since computational inexpensive, the log‐normal distribution [34, 35]. The probability density function (PDF) for a log‐normal distribution of molar masses is given by:

(1) $$f\left(M;\mu ,\sigma \right)=\frac{1}{M\sigma \sqrt{2\pi }}\exp\left(-\frac{{\left(\ln\left(M\right)-\mu \right)}^{2}}{2\sigma^{2}}\right)$$

The challenge lies in determining the appropriate internal model parameters (μ and σ for the log‐normal model) that will produce a distribution whose moments precisely match the target experimental values ($M_{n,\mathrm{target}}$ and $M_{w,\mathrm{target}}$). To solve this inverse problem, we employ a Newton‐like iterative refinement process. This optimization loop begins with an initial guess for the model parameters (μ,σ) and performs the following steps (SI for a more detailed description):

1. A discrete MMD is generated from the statistical model using the current parameters.
2. The number‐average ($M_{n,\mathrm{sim}}$) and weight‐average ($M_{w,\mathrm{sim}}$) molar masses are calculated from this simulated distribution.
3. The simulated moments are compared to the target experimental moments, and an error is calculated.
4. A numerical optimization algorithm adjusts the parameters (μ,σ) to minimize this error.
5. The parameters (μ,σ) are adjusted using specific update rules to minimize this error: μ is updated to correct the scaling of the entire distribution (primarily affecting $M_{n,sim}$), while σ is updated to correct the width of the distribution (primarily affecting the ratio $M_{w,sim}/M_{n,sim}$).

This process is repeated until the first and second statistical moments of the simulated molar mass distribution–corresponding to the number‐average (*M_n_*) and weight‐average (*M_w_*) molar masses–converge to the target values with high precision (e.g., <0.5%). To make the simulations more accurately representing experimental measurements, broadening effects, asymmetry, noise and baseline shifts can also be incorporated if the generated data is used together with experimental data having these characteristics.

Once a validated MMD is generated, it is treated as a probability density function from which a representative set of target molar masses are sampled. This sampling creates an ensemble of N discrete molar masses, $\left\{M_{1},M_{2},\text{…},M_{N}\right\}$, that collectively mirror the full distribution. This sampling can be performed in two ways: By number, where the probability of drawing a mass M_i_ is proportional to its number fraction, giving preference to the more numerous smaller chains; or by weight, where the probability is proportional to its weight fraction, favoring the heavier chains that contribute more to the overall mass. For the experiments reported herein, number‐based sampling was utilized. In general, mass‐based sampling should be preferred for macroscopic properties such as glass transition temperature, as high molecular weight chains have a disproportionate influence on these properties [23]. For our datasets, which either contain no mass data or measurements with general lower dispersity, we found no detectable difference between both sampling methods and for some data only *M_n_* is reported; therefore, a number‐based sampling was applied in the current study. But for other datasets the effect might be significant, which is why both sampling methods are available. For each of these N sampled masses, a corresponding polymer graph is subsequently generated by the kinetic Monte Carlo simulation (described in Section 2.3), ensuring that the ensemble of graphs fed to the GCN is a true and statistically sound representation of the polymer sample.

### 2.3 Graph Generation Via Optimized Kinetic Monte Carlo (kMC) Simulation

Once a representative set of target molar masses has been sampled from the MMD, the corresponding polymer graphs for each target mass have to be created. This requires a method capable of producing topologically realistic structures that reflect the underlying polymerization chemistry. To achieve this, our framework employs a physically‐grounded kinetic Monte Carlo (kMC) simulation, incorporating the underlying reactions that lead to the target polymers [36, 37]. This approach provides a versatile and extensible method for creating polymer structures by stochastically simulating the sequence of elementary reaction events based on their intrinsic kinetics. A key advantage of our implementation is its declarative nature; the simulation is governed by a user‐defined set of rules rather than a hardcoded procedure, making it adaptable to a wide range of polymerization mechanisms, from chain‐ and step‐growth to more complex branching and network formations.

To initialize a simulation, a comprehensive recipe is defined, specifying the system's components and their reactive behaviors. This recipe includes:

1. **Monomer definitions**: Each distinct monomer type is defined by its chemical structure (from which its molar mass is derived), its initial population count, and a list of its constituent reactive sites. Each site is characterized by a type (e.g., *Radical*, *Vinyl_group_head*, *Hydroxyl*) and an initial status (e.g., *activ*e, *dormant*). These sites are the fundamental units that participate in reactions.

2. **Reaction schemas**: The rules of polymerization are encoded in a set of reaction schemas. Each schema defines a reaction between a pair of site types (e.g., a *Radical* site reacting with a *Vinyl_group_head* site), its intrinsic rate constant ($k_{j}$), and the consequences of the reaction. A crucial feature is the *activation_map*, which specifies how a reaction can change the status of other, previously *dormant* sites on a reactant monomer. For example, in radical polymerization, the reaction of an *active Radical* site with a *dormant Vinyl_group_head* activates a *Vinyl_group_tail* site on the same monomer, converting it into a new *active Radical* site, thereby propagating the chain.

The simulation engine employs the Gillespie algorithm, a statistically exact method for simulating the time evolution of a well‐stirred system of chemical reactions [38]. At each step, the process first calculates the propensity, $a_{j}$, for every possible reaction channel j. The propensity (reaction probability rate) represents the probability of that reaction occurring in the next infinitesimal time step and is a function of the rate constant and the current number of available reactants.

(2) $$a_{j}=k_{j}\cdot h\left(N_{1},N_{2},\text{…}\right)$$

Here, $k_{j}$ is the intrinsic rate constant for reaction j, and the function h is a combinatorial term that accounts for the number of distinct reactant combinations. For a bimolecular reaction between species A and B, $h=N_{A}\;N_{B}$; for a self‐reaction of species A, $h=N_{A}\;\left(N_{A}-1\right)/2$. The total propensity of the system, $A_{0}$, is the sum over all possible reactions and dictates the overall rate of events.

(3) $$A_{0}=\sum_{j}a_{j}$$

The time to the next reaction, τ, is then sampled from an exponential distribution, ensuring that events occur more frequently when the total propensity is high.

(4) $$\tau =\frac{1}{A_{0}}\ln\left(\frac{1}{r_{1}}\right)$$

Here, $r_{1}\in \left(0,1\right]$ is a uniform random number. A second random number, $r_{2}$, is applied to select which specific reaction channel j will occur, with the probability of selection being proportional to its relative propensity, $a_{j}/A_{0}$. The system's state is then updated: The chosen reactants are consumed, a new covalent bond (an edge in the graph) is formed between them, and the statuses of relevant reactive sites are updated according to the reaction's *`activation_map`*.

While the kMC simulation is powerful, directly running it to generate a polymer of a target molar mass, $M_{\mathrm{target}}$, is computationally intractable. The stochastic nature of the process means that a single run with a fixed set of initial conditions will yield a distribution of polymer masses, making it virtually impossible to hit a specific target mass on demand.

The simulation can be run with a very large number of virtual particles N, increasing the statistical likelihood that the desired molecular graphs will be generated within a single run. This approach is only efficient if many different graphs of the same polymerization system are required, in which case a single simulation can yield a broad diversity of outcomes. However, if only a few specific graphs are needed, this method is not optimal, as the simulation time typically scales as $O(n^{2})$, making large‐scale simulations computationally expensive.

To address this significant challenge, we incorporated an automated outer‐loop optimization that reframes the problem as an inverse‐design task.

Bayesian optimization is used to efficiently search for the simulation parameter space, θ, to find conditions that reliably generate polymers near a desired $M_{\mathrm{target}}$. The parameter space θ is explicitly defined by the polymerization scheme, for example, the initial counts of initiator and monomer particles, and the rate constants for the initiation and propagation reactions.

For each trial in the optimization, a fast, small‐scale kMC simulation is run (e.g., with N<$10^{4}$) initial particles), and an objective function is evaluated. Crucially, rather than targeting the mass of a single, stochastically produced largest polymer, our objective function is designed for greater robustness. We calculate the number‐average molar mass ($M_{n,\mathrm{sim}}$) of the full polymer distribution produced in the small‐scale simulation and aim to minimize the absolute error relative to the target mass:

(5) $$\underset{\theta }{minimize}\left|M_{\mathrm{sim}}\left(\theta \right)-M_{\mathrm{target}}\right|$$

This approach is more stable because $M_{n,\mathrm{sim}}$ is a statistical property of the entire simulated ensemble, making it a less noisy proxy for the central tendency of the polymerization outcome compared to the mass of a single chain. By optimizing for conditions that yield an $M_{n,\mathrm{sim}}$ close to $M_{\mathrm{target}}$, we significantly increase the probability that the resulting polymer mass distribution will contain polymer graphs within the desired mass range.

Bayesian optimization is particularly well‐suited for this task as it intelligently explores the parameter space, building a probabilistic model of the objective function to decide which parameters to try next, making it highly efficient for a problem where each evaluation (a kMC run) has a non‐trivial computational cost [39].

The graphs generated during the optimization process are also usable for the sampling process, so the optimization can stop early if the required graphs are sampled.

Once the optimizer converges on a suitable parameter set, θ, and not enough graphs are already sampled, a final, large‐scale kMC simulation is executed with these optimized parameters and a much larger number of initial particles (e.g., $N>10^{5}$) to generate the final polymer graphs. This on‐demand, optimized process is repeated for each mass sampled from the MMD (if not generated by a previous kMC‐simulation), yielding a statistically representative ensemble of graphs for a single experimental data point.

It is important to acknowledge that this kMC based graph‐generation operates under several idealizations for computational tractability. The simulation assumes a well‐stirred system, neglecting diffusion limitations, the Trommsdorff (gel) effect at high conversion, and steric hindrance from polymer coiling which are factors in high concentration or bulk polymerizations [40, 41]. Incorporating these effects is highly complex as it has to incorporate reactor dimensions, internal heat transfer, solvent effects, flow simulations and probably has to be individually calibrated for each reactor setup separately. The potential gain for the use case described in this publication is also limited, since the polymer chains are not sampled directly from the resulting coarse distribution generated in the simulation, which acts as a topological‐assembler, but from experimental or simulated molar mass distributions, which can incorporate symmetric or asymmetric broadening much more easily without having to fully simulate the underlying effects. Furthermore, the reactivity of a site is considered intrinsic to its type, independent of the length or topology of the chain to which it is attached. These idealizations mean the simulation does not perfectly replicate experimental MMDs from first principles alone. This fundamental limitation reinforces our hybrid approach: We leverage the kMC simulation to generate physically plausible topologies, but we constrain the distribution of these topologies by sampling the target masses directly from realistic MMDs, thereby combining the strengths of both methods.

### 2.4 Multi‐Faceted Model Input

The methodologies described in the preceding sections culminate in the construction of a rich, multi‐faceted input designed to provide our model with a holistic understanding of a polymer sample. A central tenet of our work is that representing a polymer sample with a single, average‐property graph is fundamentally flawed, as it discards the critical information contained within the statistical distribution of structures. Our approach rectifies this by feeding the model a synergistic combination of topological, statistical, and contextual information for each experimental data point. This final input package consists of three distinct streams, which are processed by the network to learn complex structure‐property relationships.

#### 2.4.1 The Ensemble of Representative Graphs

Instead of a single graph, the primary input to our model is an ensemble of representative polymer graphs. This ensemble is generated by first sampling a set of N target molar masses from the full MMD (as described in Section 2.2) and then generating a unique, topologically realistic polymer graph for each of these target masses using the optimized kMC framework (detailed in Section 2.3). It ensures the model does not learn from a single, idealized chain but from a population of structures that mirrors the sample's dispersity. By being exposed to a variety of chain lengths and their corresponding topologies (e.g., different numbers of branches or end‐groups), the model is forced to learn features that are robust to the inherent structural variance of the polymer sample, leading to more generalizable and physically meaningful predictions.

#### 2.4.2 The Full Molar Mass Distribution Histogram

While the graph ensemble provides a stochastic representation of the MMD, we also provide the model with a direct, deterministic overview of the entire distribution. The full MMD curve, whether sourced from experimental data or generated via a statistical model, is discretized into a fixed‐size vector (i.e. a histogram). This histogram is passed to the model as an additional global feature. This input stream is complementary to the graph ensemble; while the ensemble captures specific topological instances, the histogram provides a complete summary of the sample's statistical profile, including fine details about the distribution's shape, skewness, or modality that might not be fully captured by a finite sample of graphs. This enables the model to directly correlate the overall shape of the MMD with the target property, independent of the specific graph instances it sees.

#### 2.4.3 The Contextual Feature Vector

Polymer properties are not solely a function of their chemical structure and molar mass distribution; they are also highly dependent on the external conditions under which they are measured or processed, for example, additives strongly influence mechanical properties, the addition of ions can have a profound effect on solubility or crystallinity can be influence by the processing including the temperature profile during cooling [42, 43, 44]. To account for this, our model accepts a third stream of input: A numerical and/or categorical feature vector containing relevant contextual parameters. This vector can include any available metadata associated with the experimental measurement, such as: Thermodynamic conditions (temperature, pressure), sample environment (solvent type, pH‐value) or compositional data (concentration of additives, plasticizers, or fillers).

By including this information, the model can learn the influence of these external variables and uncover potential cross‐correlations between them and the polymer's structural features. This enables more nuanced predictions and provides a mechanism to model property changes as a function of processing or experimental conditions, moving beyond a purely structure‐based prediction.

### 2.5 Structure‐Aware Graph Convolutional Network (GCN) Architecture

**FIGURE 3:**
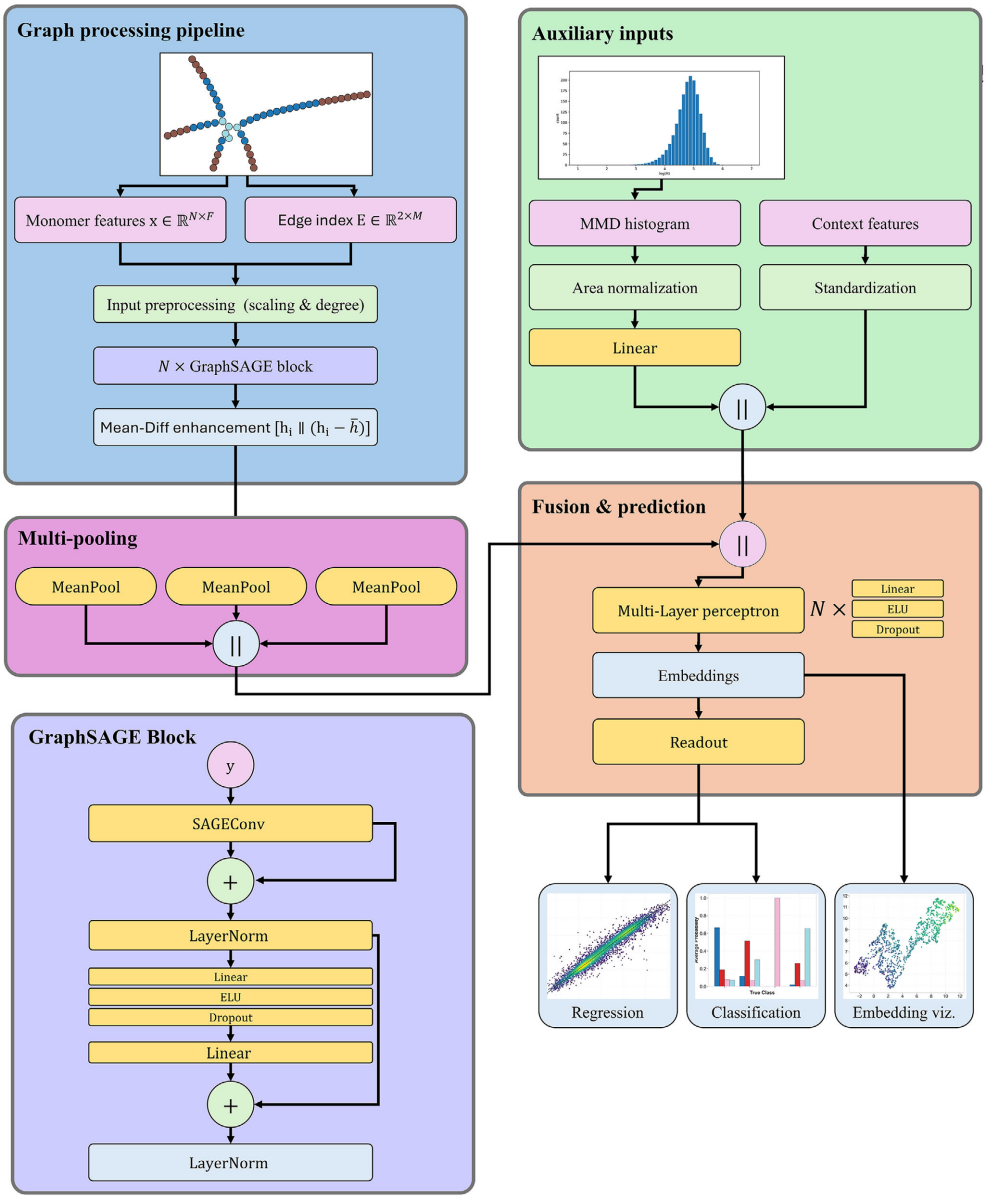
Structure‐aware graph convolutional network architecture for polymer property prediction. The model integrates three input streams: Hierarchical polymer graphs processed through GraphSAGE blocks (purple) with mean‐difference enhancement (blue), molar mass distribution histograms with linear reduction (green), and standardized contextual features. Enhanced node representations undergo multi‐perspective pooling (mean, max, sum; pink) before concatenation (||) and processing through multi‐layer perceptrons for task‐specific prediction (orange). This architecture enables learning from statistical ensembles of polymer structures while incorporating molecular mass distributions and experimental context, addressing the fundamental challenge of representing polymers as statistical systems rather than fixed molecular structures.

The predictive model is a multi‐input GCN designed to process and synthesize the hierarchical, statistical, and contextual information inherent to polymer systems. Its architecture systematically learns rich representations of local chemical environments at the monomer level, aggregates these representations into global graph embeddings, and finally merges them with statistical and contextual data to yield predictive outcomes. The data processing pipeline of the model consists of the following sequential stages (Figure 3):

#### 2.5.1 Input Processing and Feature Augmentation

Initially, diverse inputs for the network are prepared. All continuous input features–those describing monomers and ancillary contextual data–undergo feature‐wise standardization (z‐score normalization). This normalization scales each feature to have zero mean and unit standard deviation based on the training data, ensuring numerical stability and comparable contribution from each input variable during model training.

Next, the initial feature vector for each monomer node, $x_{i}$, is augmented with fundamental topological information. Specifically, the degree of each node (the number of covalent bonds for forming the graph) is calculated and concatenated to its feature vector. This augmented vector is then projected into the model's hidden dimensional space via a single, learnable linear transformation, generating an initial latent representation, $h_{i}^{\left(0\right)}$, combining intrinsic chemical properties and essential connectivity information.

#### 2.5.2 Iterative Graph Message Passing via GraphSAGE

The core of graph representation learning comprises multiple graph convolutional blocks, explicitly employing the *GraphSAGE* (Graph Sample and Aggregate) convolutional operator for message passing [45]. The node update rule at layer l is given by:

(6) $$h_{i}^{\left(l+1\right)}=W_{1}^{\left(l\right)}h_{i}^{\left(l\right)}+W_{2}^{\left(l\right)}\cdot \mathrm{mea}n_{j\in N\left(i\right)}h_{j}$$

This update consists of two components. The first part is the self‐representation, where a linear transformation ($W_{1}^{\left(l\right)}$) is directly applied to the node's own features, preserving self‐information and serving as a skip connection. The second part is the neighborhood aggregation, where the information from neighboring nodes (N(i)) is aggregated by computing the mean of their feature vectors, subsequently transformed via a separate learnable matrix ($W_{2}^{\left(l\right)}$).

Each *GraphSAGE*‐based convolutional block also integrates additional stabilizing mechanisms:

- *Residual connections*: The block input $h_{i}^{\left(l\right)}$ is added to its output to mitigate gradient vanishing issues and facilitate deep network training.
- *Layer normalization*: Applied after each residual connection to stabilize activation distributions.
- *Feed‐forward network*: After normalization, the data passes through a two‐layer fully connected sub‐network with exponential linear unit (ELU) activations and dropout for regularization. A final residual connection and another layer normalization step conclude each convolutional block.

*GraphSAGE* based convolution was chosen in contrast to other methods, such as *GCN*, *GAT*, or *GIN* as it is capable of learning inductive graph representations of the graphs for better generalizability, while staying computationally efficient [46, 47, 48, 49]. Furthermore *GraphSAGE* shows the best trade‐off between graph classification tasks fur structure awareness and regression task for the task in this work.

#### 2.5.3 Global‐Context‐Aware Feature Enhancement

Following the final graph convolution layer, a specialized contextual feature enhancement block makes each node explicitly aware of its context within its polymer graph. A global average node feature vector $\bar{h_{graph}}$ is computed by averaging all final node embeddings of the same graph. Subsequently, a difference vector is calculated for each node i:

(7) $$d_{i}=h_{i}-\bar{h_{\mathrm{graph}}}$$

This vector captures deviations of individual node embeddings from their graph‐level mean composition. It is concatenated with the original node embedding $\left[h_{i}∥d_{i}\right]$ and processed through a final learned linear transformation to produce the enhanced node embedding, $h_{i}^{\prime }$. This step implicitly contextualizes each monomer relative to the polymer's average chemical identity.

#### 2.5.4 Multi‐Faceted Graph Pooling

To achieve a fixed‐size representation for an entire polymer graph, enhanced node‐level embeddings ($h_{i}^{\prime }$) undergo multi‐faceted pooling operations. Specifically, several pooling methods (mean, maximum, and sum) are applied concurrently, each resulting in a distinct graph‐level vector. Each pooling‐derived embedding is independently refined through dedicated linear layers, normalization, and activation sub‐networks. Outputs from these parallel pooling streams are concatenated into a comprehensive, multi‐perspective embedding ($z_{\mathrm{graph}}$), encapsulating diverse graph topology and chemical composition aspects. The individual pooling methods can be selected via the model configuration and are hyperparameters, which can be dynamically changed during hyperparameter tuning. In general, having multiple different methods can lead to better outcomes as it allows for capturing different aspects of the graphs and thus generating a more holistic representation of the graph [50].

#### 2.5.5 Information Fusion and Final Prediction

In the final prediction stage, the graph embedding ($z_{\mathrm{graph}}$) is fused with embeddings from two additional input streams:

- *MMD embedding*: A low‐dimensional embedding ($z_{\mathrm{MMD}}$) of the molar mass distribution histogram, produced through a dense linear transformation, captures essential statistical shape features.
- *Contextual features*: Standardized ancillary contextual feature vector ($z_{\mathrm{context}}$) is directly incorporated.

The final concatenated vector is:

(8) $$z_{\mathrm{final}}=\left[z_{\mathrm{graph}\;}\left|\left|z_{\mathrm{MWD}}\right|\right|z_{\mathrm{context}\;}\right]$$

This integrated representation encapsulates the polymer's topological characteristics, molecular dispersity, and experimental conditions, providing a holistic input for a concluding multi‐layer perceptron (MLP) equipped with dropout for robust prediction.

For regression tasks, the model explicitly estimates predictive uncertainty by outputting parameters of a Gaussian distribution for each property–mean (μ) and logarithmic variance ($\log(\sigma^{2})$)–trained by minimizing the Gaussian Negative Log‐Likelihood loss. This simultaneously ensures accurate predictive means and calibrated confidence intervals.

For classification tasks, the final MLP outputs logits representing class scores, trained using a standard cross‐entropy loss.

## 3 Results and Discussion

### 3.1 Foundational Validation: Classification of Polymer Architecture and Sequence

A comprehensive synthetic dataset was generated to serve as a testbed for the model's core competence to learn the overall polymeric topology.

To ensure that the model learned exclusively from topological and sequential patterns, rather than from latent chemical information embedded within monomer features, each distinct monomer type was represented by a unique but randomly generated feature vector. This design choice intentionally removes any influence from the specific chemical identities of the repeating units, thereby forcing the model to deduce structural characteristics solely from the graph's connectivity and the arrangement of these abstract node types.

Using the kinetic Monte Carlo framework described in the methodology, we produced a dataset comprising hundreds of unique polymer ensembles each with 20 to 50 sampled graph representation (Supporting Information for visual representations). These ensembles were designed to span four distinct macroscopic architectures (linear, star, branching, and cross‐linked) and four different monomer sequence types (homopolymer, random copolymer, block copolymer, and gradient copolymer) with random monomer features. The model was then tasked with a multi‐label classification problem: To predict both the correct architecture and the correct sequence type for each polymer graph sampled from an ensemble. This setup directly tests whether the GCN can extract both high‐level topological information and more subtle patterns of monomer arrangement from the graph representation.

The model demonstrated exceptional proficiency in identifying the overarching polymer architecture, achieving an overall accuracy of 98.7% on the test set. The classification report reveals consistently high performance across all architectural classes, with F1‐scores of 0.99, 0.99, 0.98, and 0.98 for linear, star, cross‐linked, and branching polymers, respectively. This result indicates that the GCN is effectively learning to recognize the distinct topological signatures of each architecture.

**FIGURE 4:**
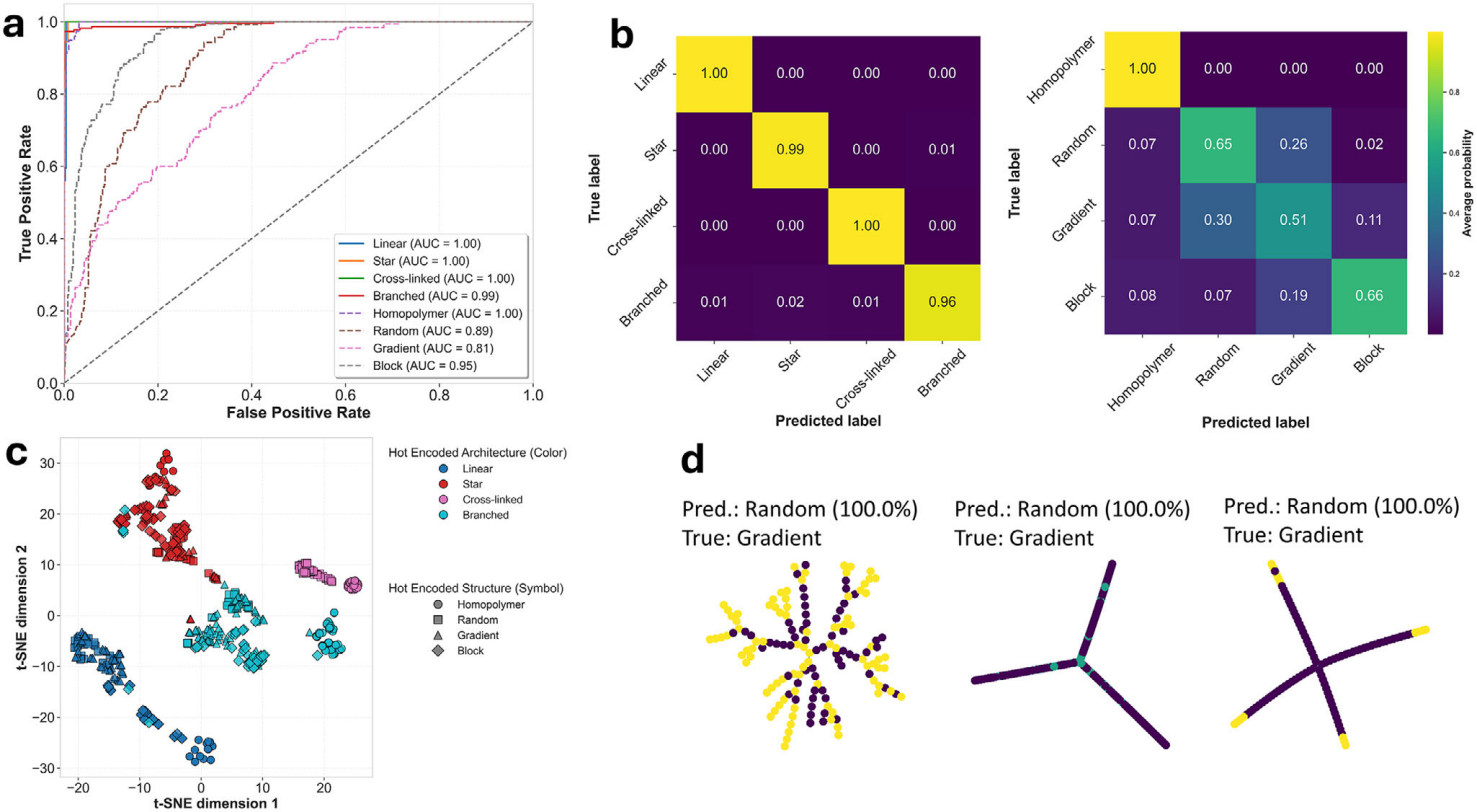
(a) Receiver Operating Characteristic (ROC) curves with corresponding Area Under the Curve (AUC) values for the multi‐label classification task. The model achieves near‐perfect discrimination for all architectural classes (AUC ≥ 0.99) and for homopolymers (AUC = 1.00), indicating a strong ability to learn these fundamental features. Performance is more nuanced for other sequence types, with lower AUC values for random (0.89 ± 0.01) and gradient (0.81) copolymers. (b) Confusion matrices displaying the model's predictive accuracy for architecture (left) and sequence (right). The architecture classification is highly accurate, with diagonal probabilities exceeding 0.96. The sequence classification matrix highlights the primary challenge: While homopolymers are perfectly identified, there is significant confusion between random, gradient, and block copolymers, with only 51% of gradient polymers correctly classified. (c) A t‐SNE visualization of the final graph embeddings, providing insight into the learned feature space. Points are colored by architecture and shaped by sequence type. The distinct clustering by color confirms that the model effectively separates graphs based on their high‐level topology (architecture). The intermixing of shapes within these clusters demonstrates the greater difficulty in distinguishing between the more subtle patterns of monomer sequence. (d) Visual examples of misclassified gradient polymers. These stochastically generated graphs, while labeled as Gradient, are predicted with high confidence to be Random or Block due to their structural resemblance to these other classes, visually explaining the confusion observed in panel (b) and highlighting the grey area between the hard labels of sequence patterns.

The success can be attributed to the message‐passing mechanism, which is inherently sensitive to graph‐theoretic properties. For instance, the model can differentiate star polymers by identifying a central node with a high degree, distinguish cross‐linked networks by the prevalence of nodes with degrees greater than two and the presence of numerous cycles, and recognize linear chains by their sequence of degree‐two nodes capped by degree‐one end‐groups. The ability to robustly differentiate these fundamental topologies from the graph structure alone is a critical validation of the hierarchical, monomer‐based graph representation. Visual inspection of a t‐SNE projection of the final graph embeddings, colored by true architectural class, would further corroborate this finding, showing distinct, well‐separated clusters for each architecture (Figure 4).

In contrast, the more subtle task of classifying the monomer sequence revealed a more nuanced performance profile, highlighting both the capabilities and the current limitations of the model. The overall accuracy for this task was 72%, with significant variation between classes.

The model identified homopolymers with near‐perfect accuracy (F1‐score of 0.91), an expected result as the uniformity of node features in a homopolymer graph presents a simple and distinct pattern.

Performance on random copolymers (F1‐score of 0.62) and block copolymers (F1‐score of 0.74) was moderate. The confusion between these classes is chemically intuitive, as a random copolymer with some statistical clustering can topologically resemble a short or imperfect block copolymer.

The most significant challenge for the model was the classification of gradient copolymers, which achieved an F1‐score of only 0.54. A closer analysis revealed that the poorest performing subclass was specifically non‐linear gradient polymers. This difficulty stems not from an inherent inability of the model to recognize gradients, but from the stochastic nature of the graph generation process itself. Gradient structures were created in the kMC simulation by assigning different, randomly selected reactivities between monomer types. If reactivities are nearly identical, the outcome is a random copolymer; if they are vastly different, the result approaches a (quasi‐)block copolymer. While the reactivities were constrained to prevent trivial outcomes (e.g., no reactivity could be less than 10% of the total), the stochastic generation can still produce individual graphs labeled as *gradient* that structurally overlap with other classes.

For instance, a set of reactivities may result in a gradient with minimal structural influence that exhibits a gradual rate of compositional change, making it nearly indistinguishable from a random copolymer, or a gradient with pronounced structural influence that shows a steep rate of compositional change, strongly resembling a block copolymer. This uncertainty is compounded in non‐linear architecture; a gradient coming from the center of a star or branched polymer can appear to the model like a random or block structure, in particular if the gradient is not pronounced. This apparent confusion does not necessarily represent a limitation of the model's discriminative power. Instead, it may reflect a physically meaningful reality: A gradient polymer whose structural composition change along the backbone is so weakly or strongly pronounced that it has a significant topological overlap with another class can be assumed to behave similarly from a physical properties’ standpoint. The model, therefore, learns to place these structurally ambiguous polymers close together in its embedding space.

### 3.2 Application to Experimental Property Prediction: Glass Transition Temperature

Having established the model's foundational capabilities on synthetic data, its performance was evaluated on the real‐world task of predicting the glass transition temperature (*T*_g_). For this purpose, a large dataset of 7,874 experimental measurements was compiled from an existing curated source [8]. This dataset consists of *T_g_* values for linear homopolymers, but no information about the molar mass distribution, which is a common limitation in publicly accessible large‐scale polymer data compilations.

To address this, the experiment was explicitly framed to validate the model's ability to learn property‐structure relationships from chemical topology alone, with the MMD input serving as a controlled, constant placeholder. To maintain architectural consistency for a subsequent fine‐tuning stage that includes MMD data, a single, uniform MMD with an *M_n_* of 50 000 g mol^−1^ and a dispersity of 1.4 was supplied for every data point (values are arbitrary picked). These values were chosen as they represent a typical polymer sample, thereby ensuring that masses associated with this placeholder input would not require excessively large updates during fine‐tuning, preserving the pre‐trained knowledge from the polymer graph representations.

For the individual entries in the dataset multiple datapoints are collected from their respective sources. These sources sometimes strongly disagree with up to >400 K in difference. It is not stated if this disagreement is from an error in the measurement or due to the fact, that *T_g_* can heavily depend on the molar mass and the processing of the polymers, reflecting experimental variability and sensitivity to unreported parameters like molar mass and dispersity [51, 52, 53]. Analysis of this intrinsic uncertainty reveals a baseline measurement‐weighted mean absolute error of approximately 10 K within the dataset itself, which represents an irreducible error floor for any predictive model (see Supporting Information for more details).

Our GCN‐model was trained on 80% of the polymer structures, with the remaining 20% reserved for validation and testing in a 5‐fold cross‐validation setting. On the held‐out test set, the model achieved predictive performance, with a Mean Absolute Error (MAE) of 26.24 ± 0.81 K and a coefficient of determination (R^2^) of 0.89 ± 0.01. This highlights the model's ability to learn complex structure‐property relationships from chemical features and graph topology alone. The assumption of a fixed MMD simplifies the learning task by removing chain length as a variable, thereby isolating the contribution of the chemical structure. This result not only underscores the model's capacity to extract salient topological features but also sets the stage for the critical fine‐tuning experiment in the following section, which directly tests the model's ability to incorporate the physically significant influence of varied molecular mass distributions.

**FIGURE 5:**
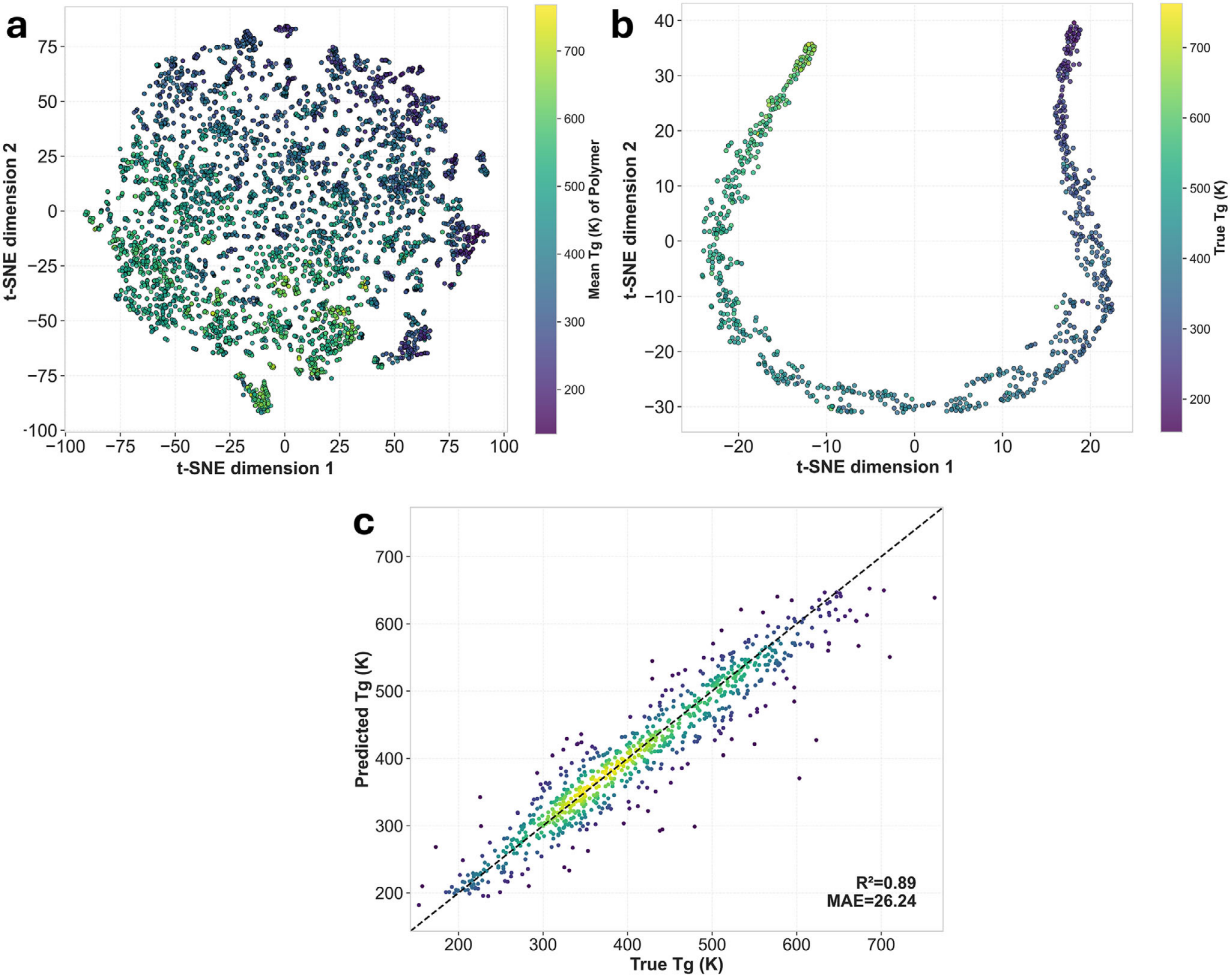
Model performance and learned feature representations for the prediction of experimental *T*_g_. (a) A t‐SNE visualization of the initial monomer feature vectors for each homopolymer in the test set, colored by their experimental *T*_g_‐value. The lack of any discernible pattern indicates that the input features are not trivially correlated with the target property. (b) A t‐SNE visualization of the final graph embeddings generated by the GCN, immediately before the final prediction layer. The model has successfully organized the polymers into a highly structured, continuous manifold where *T*_g_‐values are smoothly ordered, demonstrating that it has learned a meaningful structure‐property relationship. (c) Parity plot comparing the model's predicted *T*_g_‐value against the true experimental values for the held‐out test set. The strong clustering of points around the ideal line (y = x, dashed) signifies high predictive accuracy, achieving an R^2^ of 0.89 ± 0.01 and a Mean Absolute Error (MAE) of 26.24 ± 0.81 K. The density‐based coloring (yellow = high, purple = low) shows the majority of datapoints are close to an ideal prediction.

A visualization of the initial monomer feature vectors – generated via polyBERT featurization of the respective PSMILES [11] – demonstrates that they provide a powerful starting point for property prediction (Figure 5a). The t‐SNE projection is not random; instead, it shows significant local structure and a discernible, albeit complex, relationship with the *T*_g_‐value. This inherent organization confirms that the polyBERT embeddings capture nuanced chemical information relevant to the *T*_g_‐value. However, to achieve high‐fidelity predictions, this rich monomer‐level information must be integrated across the entire polymer graph; especially if more complex structure, not just linear homopolymers are involved; which is the essential role of our GCN architecture.

In stark contrast, a visualization of the final graph embeddings from the model's penultimate layer shows a remarkable transformation (Figure 5b). The model has learned to organize the polymers into a highly structured, low‐dimensional manifold where the *T*_g_‐value varies smoothly and continuously along the principal axes of the embedding space.

This learned, property‐centric representation directly translates into the high predictive accuracy demonstrated in the parity plot (Figure 5c), which shows strong correlation between predicted and true *T*_g_‐values, with data points tightly clustered around the ideal y = x line. The model's MAE of 26.24 ± 0.81 K represents a robust result given the significant documented disagreements and missing statistical information (e.g., MMD) in the source data.

For this specific dataset, which contains only linear homopolymers, a comparison to baseline models, which can work with the PolyBERT embeddings directly (linear regression, random forest, support vector regression) shows significantly lower performance (MAE > 33 K) [11]. Furthermore, these baselines cannot capture any copolymer information or polymer architectures, making them only comparable for the simple case of a linear homopolymer–see Supporting Information for further details.

### 3.3 Fine‐Tuning to Capture Molar Mass Dependence of Glass Transition Temperature

While the model demonstrated robust performance on the large‐scale *T*_g_‐dataset, a significant limitation of that data is the general absence of molar mass information for the polymer samples. Consequently, the model pre‐trained on this data could not learn the well‐established physical relationship between a polymer's chain length and its *T*_g_‐value. To address this gap and to assess the model's ability to learn more subtle, physically grounded trends, a fine‐tuning experiment was conducted using a smaller, high‐fidelity in‐house dataset. This dataset was specifically curated to include both homopolymers and copolymers with experimentally measured *T*_g_‐values and their corresponding molar mass distributions (140 entries with a full MMD from SEC measurements and 331 as *M_n_*/*M_w_*—pairs) [21, 54, 55, 56].

**FIGURE 6:**
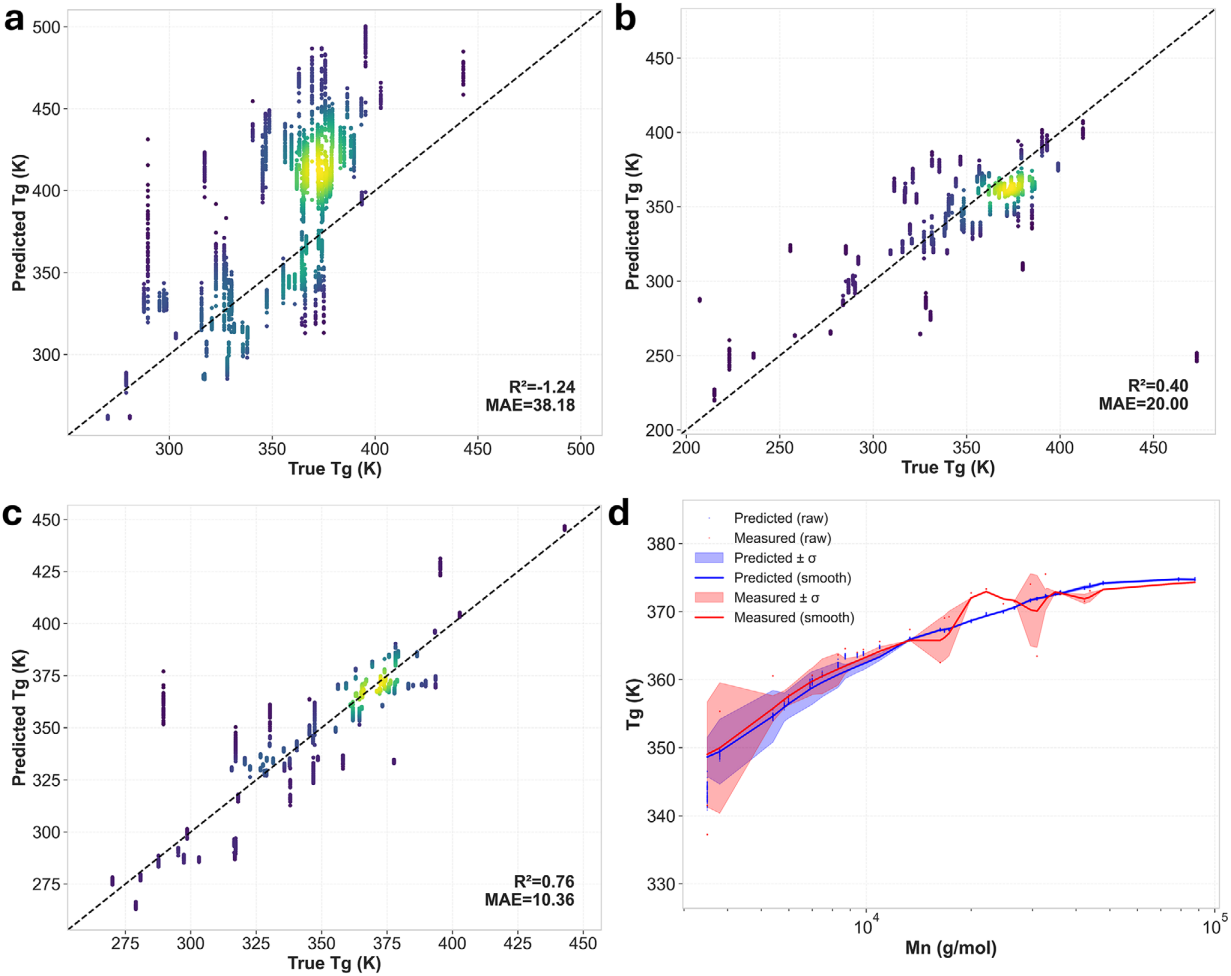
Fine‐tuning the GCN model to capture the molar dependence of the *T*_g_‐value. (a) Parity plot showing the performance of the initial, pre‐trained model on the high‐fidelity dataset. The model fails to make meaningful predictions (R^2^ = −1.24, MAE = 38.18 K) because it was not trained on molar mass data. (b) Parity plot of the same dataset after artificially setting the molecular mass distribution information for all datapoints to be the identical to the MMD from the dataset of the pretrained model (R^2^ = 0.54, MAE = 17.58 K). This shows that a high contribution to the error in (a) is a result of the newly present variance in the MMD input. Furthermore, the new dataset contains copolymers, which are also not present in the previous dataset, which also contributes to the error. (c) Parity plot of the same dataset after fine‐tuning the model for one epoch with molecular mass distribution information. The performance dramatically improves (R^2^ = 0.76 ± 0.06, MAE = 10.36 ± 2.5 K), demonstrating that the model successfully learned to incorporate the influence of molar mass and copolymer structures. (c) A comparison of experimental data (red) and model predictions (blue) for the *T_g_*‐value of polystyrene as a function of number‐average molar mass (*M*_n_). The fine‐tuned model accurately reproduces the known physical trend, including the asymptotic plateau at high molar masses, confirming it has learned an interpretable, physically grounded relationship. Shaded regions represent the standard deviation of the measured data and the predicted uncertainty from the model.

First, the pre‐trained model was applied directly to this new dataset without any fine‐tuning. As expected, its performance was poor, yielding a Mean Absolute Error (MAE) of 38.18 K and a negative coefficient of determination (R^2^ = −1.24), indicating that its predictions were worse than simply using the mean *T*_g_ of the dataset (Figure 6a). This result is logical: The model was trained to predict a single, average *T*_g_‐value for a given polymer chemistry, and was therefore unable to account for the large variations in the *T*_g_‐value caused by differing molar masses within this specialized dataset. Furthermore, the curated dataset contains copolymer entries, which is also new information and can be considered as a domain extension. To further investigate these factors, the dataset was modified such that all entries have the same MMD, identical to the previous dataset (*M_n_* of 50 000 g mol^−1^ and a dispersity of 1.4), resulting in better predictive performance (R^2^ = 0.54, MAE = 17.58 K). Based on these results, we assume that for the pretrained model the domain extension to copolymers is relatively well transferable.

Next, the model's weights were fine‐tuned for a single epoch using this richer dataset. The fine‐tuning process utilized the model's full multi‐faceted input architecture, providing the experimentally determined MMD as an explicit input alongside the graph representation. The improvement was immediate and dramatic. The fine‐tuned model achieved an MAE of 10.36 ± 2.5 K and an R^2^ of 0.76 ± 0.06 (Figure 6b). This significant enhancement in accuracy demonstrates that the model successfully learned to integrate the statistical MMD information with the topological graph features to make physically relevant predictions.

To verify that the model had learned a generalizable physical principle rather than simply memorizing the training data, it was used to predict the *T*_g_‐value for polystyrene, one of the most common polymers in the dataset, across a continuous range of synthetically generated molar masses. The results show that the model's predictions closely track the experimental trend, capturing the characteristic positive correlation between the *T*_g_‐value and molar mass, as well as the asymptotic plateau at high molar masses (Figure 6c). This predictive behavior is in excellent agreement with foundational principles of polymer physics, quantitatively described by theories such as the Flory–Fox equation [57].

This finding is significant for two primary reasons. First, it demonstrates that the structure‐aware GCN is not merely functioning as a black‐box pattern recognizer but is capable of learning physically interpretable, multi‐variable relationships. Second, it validates the efficacy of the model's architecture, confirming that the network can successfully integrate statistical information from the MMD with topological information from the graph. This successful application of a pre‐training and fine‐tuning workflow highlights a powerful paradigm for polymer informatics: leveraging vast but incomplete public data to build a foundational model, which can then be refined with smaller, high‐quality experimental datasets to capture complex structure‐property relationships.

## 4 Conclusion

This study introduced a novel, structure‐aware framework designed to overcome the fundamental limitations of applying conventional machine learning models for polymer science related questions. Classical methods of QSPR‐modeling, as it is used for small molecules, cannot be directly applied to polymers, as they cannot be described by a fixed molecular structure alone, which most current methods are based upon. By departing from the small‐molecule paradigm of representing materials as single structures, our approach embraces the inherent statistical and hierarchical nature of polymers. The developed methodology integrates three core components: A hierarchical graph representation where nodes signify monomer units, the explicit incorporation of full molecular mass distributions (MWDs) to account for sample dispersity, and an ensemble‐based learning strategy where the model is trained on a collection of topologically realistic graphs generated via an optimized kinetic Monte Carlo simulation. This multi‐faceted input provides the model with a holistic understanding of a polymer sample, encompassing its chemical composition, architectural diversity, statistical dispersity, and experimental context.

The efficacy of this approach was systematically validated through a series of increasingly complex tasks. On a synthetic dataset, the model demonstrated an exceptional ability to learn high‐level topological features, achieving near‐perfect classification of polymer architectures. The model also showed more nuanced discrimination of monomer sequence, successfully identifying distinct patterns while also capturing the physically meaningful ambiguities that arise from the stochastic generation of copolymer structures. When applied to a large experimental dataset, the GCN model achieved high predictive accuracy for the glass transition temperature of polymers (R^2^ = 0.89 ± 0.01), learning to map complex chemical structures to a key macroscopic property. Crucially, a fine‐tuning experiment on a high‐fidelity dataset demonstrated that the model could successfully learn the well‐established physical relationship between chain length and *T_g_*‐value. This confirmed the model's capacity to integrate statistical MMD data and learn physically interpretable, multi‐variable relationships.

The significance of this work extends beyond the immediate predictive performance, presenting a methodological paradigm shift for polymer informatics. By treating polymer samples as statistical ensembles, this framework enables more physically realistic and robust predictions. The demonstrated pre‐training and fine‐tuning workflow highlights a powerful strategy for bridging the gap between large‐scale, incomplete public data and smaller, high‐fidelity experimental datasets, which is a common challenge in materials science. This approach has the potential to accelerate the in silico screening and design of new polymers by providing more accurate property predictions that account for the critical influence of molar mass and its distribution.

Despite these promising results, several limitations and avenues for future research are acknowledged. The kMC graph generation, while physically grounded, operates under idealizations. Most importantly, the model's full potential is contingent on the availability of high‐quality data; a concerted community effort to generate and curate large datasets that include MMD's and other contextual information is essential for advancing polymer informatics. Looking forward, the representations from this model can be leveraged for inverse design, creating a generative framework capable of proposing novel polymer structures with specific, tailored property profiles, thereby accelerating the discovery of next‐generation materials.

## Funding

Thüringer Aufbaubank (VFE 1000241), Joachim Herz Foundation, Bayerisches Zentrum für Batterietechnik (BayBatt).

## Usage of Generative AI

Generative AI was used solely for language editing (grammar, spelling, and clarity) as described in the Supporting Information. It did not generate scientific content, perform calculations, or alter the authors’ interpretations, and all revisions were reviewed and approved by the authors.

## Conflicts of Interest

The authors declare no conflicts of interest.

## Supporting information




**Supporting File**: marc70190‐sup‐0001‐SuppMat.docx.

## Data Availability

The data that support the findings of this study are available in the supplementary material of this article. The packages for kinetic Monte Carlo simulation for polymer graph generation is available under https://github.com/JulianKimmig/polysim/, the package for sec reading and simulation is available under https://github.com/JulianKimmig/secanalysis/ and the model source code can be found at https://github.com/JulianKimmig/polymer_gc.
