# Supplementary material for: Structure‐Aware Machine Learning for Polymers: A Hierarchical Graph Network for Predicting Properties From Statistical Ensembles
Source: Macromol Rapid Commun. 2026 Jan 6;47(12):e00671. doi: 10.1002/marc.202500671 (PMC13309149; doi:10.1002/marc.202500671)
Supplement: Supplementary file 1 — Supporting File: marc70190‐sup‐0001‐SuppMat.docx. [file MARC-47-e00671-s001.docx]

Supporting Information

Structure-aware machine learning for polymers: A hierarchical graph network for predicting properties from statistical ensembles

Julian Kimmig, Yannik Köster, Timo Koswig, Punith Raviswamy, Subhash V. S. Ganti, Stefan Zechel, Christopher Kuenneth, Ulrich S. Schubert*

Julian Kimmig, Yannik Köster, Dr. Timo Koswig, Dr. Stefan Zechel, Prof. Dr. Ulrich S. Schubert

Laboratory of Organic and Macromolecular Chemistry (IOMC), Friedrich Schiller University Jena, Humboldstr. 10, 07743 Jena, Germany

Jena Center for Soft Matter (JCSM), Friedrich Schiller University Jena, Philosophenweg 7, 07743 Jena, Germany

ulrich.schubert@uni-jena.de

Julian Kimmig

Linkdlab GmbH, Botzstraße 5, 07743 Jena, Germany

Prof. Dr. Ulrich S. Schubert

Helmholtz Institute for Polymers in Energy Applications Jena (HIPOLE Jena)
Lessingstr. 12-14, 07743 Jena, Germany

Helmholtz-Zentrum Berlin for Materials and Energy GmbH,

Hahn-Meitner-Platz 1, 14109 Berlin, Germany

Punith Raviswamy, Subhash V. S. Ganti, Jun.-Prof. Dr. Christopher Kuenneth

Faculty of Engineering Science, University of Bayreuth, Universitätsstraße 30, 95447 Bayreuth, Germany

Subhash V. S. Ganti, Jun.-Prof. Dr. Christopher Kuenneth

Bavarian Center for Battery Technology (BayBatt), University of Bayreuth, Weiherstraße 26, 95448 Bayreuth, Germany

Table of content

S1. Detailed Methodology and Implementation 3

S2. Kinetic Monte Carlo simulation framework implementation 8

Overview of the kinetic Monte Carlo engine 8

Reaction channel classification 8

Propensity calculation and reaction selection 8

Data structures and memory management 9

Performance optimization strategies 10

Graph construction and analysis 10

Example code 11

S3. Size exclusion chromatography (SEC) simulation framework 12

Overview of SEC data generation 12

Iterative parameter refinement algorithm 13

Chromatographic realism enhancement 14

Implementation details 15

S4. Dataset analysis 16

PolyMetriX *T_g_*-dataset 16

Curated dataset by the authors 18

S5. Visualization of graphs of the synthetic polymer architecture dataset 21

Linear homopolymers 21

Linear random copolymers 21

Linear gradient copolymers 21

Linear block copolymers 22

Branching homopolymers 23

Branching random copolymers 24

Branching gradient copolymers 25

Branching block copolymers 26

Cross-linked homopolymers 27

Cross-linked random copolymers 28

Cross-linked gradient copolymers 29

Star homopolymers 30

Star random copolymers 31

Star gradient copolymers 32

Star block copolymers 33

S6. Usage of generative AI during the manuscript preparation: 34

1. Detailed Methodology and Implementation
   1. Graph convolutional network architecture specification

Overall architecture overview

The structure-aware Graph Convolutional Network (GCN) implements a multi-stage processing pipeline designed specifically for hierarchical polymer representations. The architecture consists of five primary components: (1) Input preprocessing and feature standardization, (2) iterative graph message passing *via* modified *GraphSAGE* blocks, (3) global-context-aware feature enhancement, (4) multi-faceted graph pooling operations, and (5) multi-layer perceptron (MLP) fusion and prediction layers.

Input processing and feature standardization

Feature scaling implementation

The model employs custom *StandartScaler* modules for feature-wise z-score normalization across three input streams:

- **Monomer features**: Standardization applied to the initial node feature matrix with dimensionality *monomer_features* (*e.g*., 600 for polyBERT featurization)
- **Additional contextual features**: Separate scaler for auxiliary experimental parameters with dimensionality *additional_features*
- **Target properties**: Regression-specific scaler for ground truth values with dimensionality *num_target_properties* (1 for *T*_g_-prediction)

The scaler implementation maintains numerical stability through an epsilon parameter
(ε = 1×10⁻⁸) and stores learned statistics as registered buffers for consistent inference scaling.

Initial feature augmentation

Node features undergo augmentation with topological connectivity information through degree calculation. The degree of each node is computed using PyTorch Geometric's degree function and concatenated to the original feature vector, creating an augmented input of size monomer_features + 1. This augmented representation is subsequently projected into the model's hidden dimensional space via a learnable linear transformation:

$$\begin{aligned} h_{i}^{\left( 0 \right)} = Linear(x_{i} || degree(i))\#\left( S SEQ SuppEq \backslash* MERGEFORMAT 1 \right) \end{aligned}$$

where the output dimensionality is gc_*features*.

Graph convolutional block architecture

Core GraphSAGE implementation

The graph convolution operation employs the *SAGEConv* operator from PyTorch Geometric, implementing the *GraphSAGE* aggregation scheme.^[1, 2]^ Each *GCBlock* module contains:

**Primary convolution layer:**

- *SAGEConv* layer with input and output channels both set to *gc_features*
- Learnable weight matrices *W₁* and *W₂* for self-features and neighbor aggregation respectively
- Mean aggregation function for neighborhood information

**Stabilization mechanisms:** The architecture incorporates multiple regularization and stabilization techniques:

1. **Primary residual connection**: Direct addition of input features to convolution output
2. **Layer normalization**: Applied immediately after the residual connection using *LayerNorm*
3. **Feed-forward network**: Two-layer MLP with:
   - First linear layer: *gc_features* → *gc_features*
   - ELU activation function
   - Dropout regularization
   - Second linear layer: *gc_features* → *gc_features*
4. **Secondary residual connection**: Addition of feed-forward output to normalized features
5. **Final layer normalization**: Applied to the complete block output

Sequential processing

Multiple *GraphSAGE* (default 3) blocks are applied sequentially using PyTorch Geometric's Sequential wrapper, enabling efficient batched processing across polymer graphs.

Global-context-aware feature enhancement

The *MeanDiffFeatureBlock* implements explicit global context integration through difference feature computation:

1. **Global average calculation**: Computation of graph-level mean features using *global_mean_pool*
2. **Broadcast operation**: Extension of graph-level means to all nodes within each graph *via* batch indexing
3. **Difference vector computation**: Element-wise subtraction of global mean from individual node features
4. **Feature concatenation**: Concatenation of original node features with difference vectors, doubling the feature dimensionality
5. **Dimensionality reduction**: Linear projection back to original *gc_features* dimensionality

This mechanism enables each node to maintain awareness of its deviation from the overall chemical composition of its parent polymer graph.

Multi-faceted pooling architecture

The *PoolingLayer* class provides a unified interface for different aggregation strategies, each incorporating learned transformations:

**Supported pooling operations:**

- Mean pooling: *global_mean_pool*
- Maximum pooling: *global_max_pool*
- Sum pooling: *global_add_pool*

**Per-pooling processing pipeline:** Each pooling operation outputs a graph-level vector of size *gc_features*, which undergoes:

1. Layer normalization
2. Linear transformation (*gc_features* → *gc_features*)
3. ELU activation
4. Dropout regularization
5. Second linear transformation
6. Residual connection with normalized input
7. Final layer normalization

**Multi-perspective concatenation**

If multiple pooling strategies (*n*) operate in parallel, the resulting tensors are concatenated to a combined representation of dimensionality *n* × *gc_features*

Information fusion and prediction architecture

**Input stream integration**

The final prediction module integrates three information streams:

1. **Graph embeddings**: Concatenated pooling outputs
2. **Molar mass distribution**: Reduced histogram representation *via* linear projection from *mass_distribution_buckets* to *mass_distribution_reduced* (default: 10)
3. **Additional contextual features**: Direct inclusion of standardized experimental parameters

**Multi-layer perceptron (MLP) configuration**

The fusion MLP consists of multiple sequential blocks, each implementing:

- Linear transformation maintaining input dimensionality
- ELU activation function
- Dropout regularization (rate = 0.155)

Total MLP input dimensionality: *n* × *gc_features* (pooling) + *distribution* (MMD) + *additional_input_features*.

**Output layer specifications**

For regression tasks with *logits_output* = false, a single linear layer projects from MLP output dimensionality to *num_target_properties*. The model uses Root Mean Square Error (RMSE) loss with mean reduction for training optimization.

$$\begin{aligned} ŷ = Linear(x\_mlp\mathbb{) \in R^(}batch\_size \times num\_target\_properties)\#\left( S SEQ SuppEq \backslash* MERGEFORMAT 2 \right) \end{aligned}$$

For uncertainty-aware regression (*logits_output* =True), the *LogitsOutput* module implements a probabilistic framework that models predictions as Gaussian distributions. The architecture outputs parameters for each target property distribution:

$$\begin{aligned} raw\_output = Linear(x\_mlp\mathbb{) \in R^(}batch\_size \times2\times num\_target\_properties)\#\left( S SEQ SuppEq \backslash* MERGEFORMAT 3 \right) \end{aligned}$$

$$\begin{aligned} \mû, log(\sigma^{2}) = reshape(raw\_output, [batch\_size, num\_target\_properties, 2])\#\left( S SEQ SuppEq \backslash* MERGEFORMAT 4 \right) \end{aligned}$$

The model predicts both mean values (*μ̂*) and logarithmic variance (*log(σ²)*) for each target property. This parameterization ensures numerical stability through log-space variance representation while enabling direct optimization *via* Gaussian Negative Log-Likelihood loss:

$$\begin{aligned} \hat{\sigma^{2}}=exp(min(10,max(-10, log(\sigma^{2}) )))\#\left( S SEQ SuppEq \backslash* MERGEFORMAT 5 \right) \end{aligned}$$

$$\begin{aligned} NLL = 0.5 * (log\left( \hat{\sigma^{2}} \right)+\left( y-\mu\right)^{2}/\hat{\sigma^{2}})\#\left( S SEQ SuppEq \backslash* MERGEFORMAT 6 \right) \end{aligned}$$

Logarithmic variance predictions are clamped within [-10, 10] to prevent numerical instabilities during training. During inference, the model provides both point predictions and uncertainty estimates.

For classification tasks, the architecture accommodates multi-task scenarios through a unified output layer that handles variable numbers of classes per task (in the example classification of topological structure and repeating unit sequence class).

During loss computation, logits are partitioned according to *num_classes_per_task* specifications, with each task's logits processed independently through CrossEntropyLoss. The total loss represents the arithmetic mean across all classification tasks:

$$\begin{aligned} L_{total} =\frac{\sum_{k=0}^{K} CrossEntropyLoss\left( logits_{k},y_{k} \right)}{K}\#\left( S SEQ SuppEq \backslash* MERGEFORMAT 7 \right) \end{aligned}$$

where K denotes the number of classification tasks. This design enables simultaneous prediction of multiple categorical properties (*e.g*., polymer architecture and monomer sequence classification) while maintaining balanced learning dynamics across tasks with different numbers of classes.

Optimized hyperparameter configuration

The following hyperparameters were determined *via* Optuna optimization for *T_g_*-prediction:^[3]^

| Parameter | Optimized value | Description |
| --- | --- | --- |
| *monomer_features* | 600 | Input node feature dimensionality |
| *gc_features* | 41 | Hidden layer dimensionality |
| *num_gnn_layers* | 3 | Number of *GraphSAGE* blocks |
| *mlp_layer* | 2 | Number of MLP fusion layers |
| *dropout_rate* | 0.155 | Dropout probability |
| *mass_distribution_reduced* | 8 | MMD embedding dimensionality |
| *pooling_layers* | [mean, max, sum] | Multi-perspective pooling strategy |

**Supporting Table 1**: Set of optimized hyperparameters for the model predicting T_g_ values from our curated dataset. Hyperparameters where optimized using Optuna.

1. Kinetic Monte Carlo simulation framework implementation

Overview of the kinetic Monte Carlo engine

The polymer simulation framework employs a direct implementation of the Gillespie algorithm for kinetic Monte Carlo (kMC) simulation, optimized for computational efficiency through Numba just-in-time compilation.^[4, 5]^ The core simulation engine models polymerization as a series of discrete reaction events, where each event represents the formation of a covalent bond between reactive sites on different monomer units.

The simulation proceeds through iterative time steps, with each step comprising four fundamental operations: (1) Calculation of reaction propensities for all possible reaction channels, (2) stochastic time advancement using an exponential distribution, (3) probabilistic selection of a specific reaction channel, and (4) system state update including site consumption and potential activation of dormant sites.

Reaction channel classification

The framework distinguishes two distinct reaction channel types based on the initial status of participating sites:

**Active-active (AA) channels**: Both reacting sites begin in the ACTIVE status. These reactions typically represent termination events in chain-growth polymerization or direct addition/condensation in step-growth systems.

**Active-dormant (AD) channels**: One site is ACTIVE while the other is DORMANT. These reactions commonly model propagation steps in chain-growth polymerization, where an active radical site reacts with a dormant vinyl group, subsequently activating a new radical site through the activation mapping mechanism.

Propensity calculation and reaction selection

Reaction propensities are calculated according to mass-action kinetics, where the propensity $a_{j}$ for reaction channel $j$ is given by:

$$\begin{aligned} a_{j}=k_{j} \cdot h(N_{1},N_{2}, \ldots)\#\left( S SEQ SuppEq \backslash* MERGEFORMAT 8 \right) \end{aligned}$$

where $k_{j}$ represents the intrinsic rate constant and $h$ denotes the combinatorial function accounting for the number of available reactant pairs. For bimolecular reactions between distinct species A and B, $h=N_{A}N_{B}$, while self-reactions employ $h=N_{A}\left( N_{A}-1 \right)/2$ to prevent self-interaction and account for the reduced number of unique pairs.

Time advancement follows the standard Gillespie prescription:

$$\begin{aligned} \Delta t=\frac{1}{A_{0}}\ln\left( \frac{1}{r_{1}} \right)\#\left( S SEQ SuppEq \backslash* MERGEFORMAT 9 \right) \end{aligned}$$

where $A_{0}=\sum_{j} a_{j}$ represents the total system propensity and $r_{1}$ is a uniform random number$[0,1]$.

Data structures and memory management

Hierarchical data organization

The simulation employs a hierarchical data structure optimized for computational efficiency and memory access patterns. The core data arrays include:

**Sites data array**: A two-dimensional NumPy array of shape $(N_{sites} , 4)$ storing for each site: Monomer identifier, site type identifier, current status, and intra-monomer site index.

**Monomer data array**: A mapping array of shape $\left( N_{\text{monomers}}+1, 2 \right)$ containing monomer type identifiers and offset indices into the sites array, enabling rapid iteration over sites belonging to specific monomers.

**Available sites collections**: Numba-typed dictionaries mapping site type identifiers to lists of globally indexed sites, separated by status (*ACTIVE/DORMANT*) for efficient propensity calculations.

O(1) site management

Critical to simulation performance is the maintenance of available site lists during reaction events. The framework implements an O(1) swap-and-pop removal algorithm that avoids costly array shifting operations. When a site is consumed in a reaction, it is removed from the available list by swapping its position with the last element and truncating the array. Corresponding position mapping dictionaries are updated to maintain consistency for subsequent removals.

This approach transforms site list maintenance from O(N) to O(1) complexity, representing a crucial optimization for large-scale simulations where millions of reaction events may occur.

Activation logic implementation

Post-reaction activation represents a key mechanistic feature enabling the simulation of complex polymerization processes such as radical chain growth. The activation system operates through user-defined per-reaction mapping dictionaries that specify which dormant site types transform into which active types following the reactions.

The activation algorithm searches the product monomer for the first dormant site matching the target type (excluding the site that just participated in the reaction), updates its type and status, and transfers it from the dormant to active site collections. This mechanism enables the modeling of radical transfer, chain propagation, and other activation-dependent processes.

Performance optimization strategies

Numba just-in-time compilation

The core simulation loop is implemented as a Numba-compiled function operating exclusively on NumPy arrays and Numba-compatible data structures. This compilation strategy achieves near-C performance levels while maintaining Python's development flexibility. The compilation process includes explicit type specifications for all variables and function signatures to maximize optimization effectiveness.

Vectorized data processing

Initial system setup employs vectorized NumPy operations wherever possible to minimize Python interpreter overhead. Monomer and site data population utilizes array broadcasting and concatenation operations rather than iterative construction, resulting in significant speedup for large systems.

Graph construction and analysis

NetworkX integration

Following simulation completion, the framework constructs NetworkX graph objects representing the final polymer network topology.^[6]^ Node attributes include monomer type identifiers and molar masses.

Molar mass distribution calculation

Molar mass analysis operates on connected components of the polymer graph, with each component representing an individual polymer chain or network fragment. The framework calculates both number-average (*M_n_*) and weight-average (*M_w_*) molar masses according to standard definitions:

$$\begin{aligned} M_{n}=\frac{\sum_{i} N_{i}M_{i}}{\sum_{i} N_{i}}\#\left( S SEQ SuppEq \backslash* MERGEFORMAT 10 \right) \end{aligned}$$

$$\begin{aligned} M_{w}=\frac{\sum_{i} N_{i}M_{i}^{2}}{\sum_{i} N_{i}M_{i}}\#\left( S SEQ SuppEq \backslash* MERGEFORMAT 11 \right) \end{aligned}$$

where $N_{i}$ and $M_{i}$ represent the count and molar mass of species $i$, respectively.

Example code

# Import necessary modules for simulation and analysis

from polymcsim import (

MonomerDef,

ReactionSchema,

SimParams,

Simulation,

SimulationInput,

SiteDef,

plot_chain_length_distribution,

)

# Configure the simulation input for free radical polymerization of methyl methacrylate

# This represents a canonical chain-growth polymerization system with distinct initiation,

# propagation, and termination phases characteristic of radical polymerization mechanisms

mma_radical_config = SimulationInput(

# Define the monomer population and their reactive site architecture

monomers=[

# Initiator species: Low molecular mass compounds containing labile bonds

# that decompose to generate primary radicals for chain initiation

MonomerDef(

name="Initiator", # Descriptive identifier for the initiating species

count=5000,   # Initial population: 200 initiator molecules

molar_mass=64.0, # Molecular mass in g/mol (representative of AIBN-type initiators)

sites=[

SiteDef(

type="I", # Site type identifier for initiating radical sites

status="ACTIVE"  # Initial status: immediately available for reaction

)

],

),

# Monomer species: Methyl methacrylate with vinyl functionality for chain propagation

# Each monomer contains two distinct reactive sites representing different chemical environments

MonomerDef(

name="MMA",  # Methyl methacrylate monomer designation

count=500000, # Monomer population: 20,000 units (100:1 monomer:initiator ratio)

molar_mass=100.1, # Experimental molar mass of methyl methacrylate (C5H8O2)

sites=[

SiteDef(

type="Vinyl", # Vinyl group (C=C) available for radical addition

status="DORMANT" # Initially inactive, requires radical attack for activation

),

SiteDef(

type="RadicalSite",  # Potential radical site formed after vinyl consumption

status="DORMANT" # Initially dormant, activated post-reaction via activation mapping

),

],

),

],

# Define the reaction network governing polymerization kinetics

# Each reaction channel represents a distinct mechanistic pathway with associated rate constants

reactions={

# Initiation reaction: Primary radical attacks dormant vinyl group

# This represents the rate-determining step for chain formation

frozenset(["I", "Vinyl"]): ReactionSchema(

activation_map={

"RadicalSite": "Radical" # Post-reaction activation: dormant RadicalSite becomes active Radical

},

rate=1.0, # Relative rate constant for initiation (normalized baseline)

),

# Propagation reaction: Growing radical chain attacks additional monomer units

# This reaction exhibits higher rate constant reflecting favorable thermodynamics

frozenset(["Radical", "Vinyl"]): ReactionSchema(

activation_map={

"RadicalSite": "Radical" # Chain growth continuation: new radical site formation

},

rate=200.0,  # High rate constant typical of propagation (kp >> ki)

),

# Termination reaction: Radical-radical coupling leading to chain cessation

# Represents bimolecular termination through combination mechanism

frozenset(["Radical", "Radical"]): ReactionSchema(

rate=100.0   # Moderate rate constant for diffusion-controlled termination

),

# Note: No activation_map specified as termination consumes both radicals without generating new active sites

},

# Simulation parameters controlling execution and convergence criteria

params=SimParams(

max_conversion=0.9,  # Terminate simulation at 90% monomer conversion

random_seed=42   # Reproducibility seed for stochastic simulation

),

)

# Initialize the kinetic Monte Carlo simulation engine with the configured system

# The Simulation class encapsulates the Numba-optimized Gillespie algorithm implementation

sim = Simulation(mma_radical_config)

# Execute the simulation to completion based on specified termination criteria

# Returns SimulationResult object containing the final polymer network and execution metadata

result = sim.run()

# Extract the polymer network graph and simulation metadata for subsequent analysis

# The graph represents the final topology with nodes as monomers and edges as covalent bonds

graph, _ = result.graph, result.metadata

# Generate visualization of the chain length distribution for polymer characterization

# This plot provides insight into the degree of polymerization distribution

# Save path specification enables automated figure generation for publication workflows

plot_chain_length_distribution(

graph,   # NetworkX graph object containing polymer topology

save_path= "mma_radical_polymerization_mmd.png"  # Output file path for molecular mass distribution plot

)


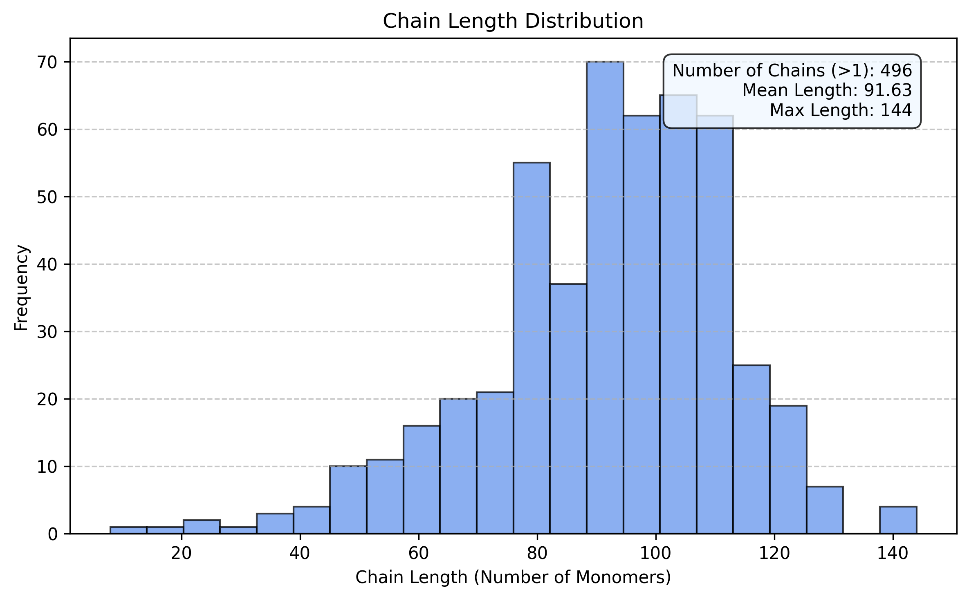


**Figure S1**: Resulting chain length distribution of the example polymerization.

1. Size exclusion chromatography (SEC) simulation framework

Overview of SEC data generation

The framework incorporates a comprehensive SEC simulation module (SimSEC) designed to generate realistic chromatographic traces that accurately reproduce experimental molar mass distributions. This simulation capability addresses the critical challenge of data scarcity in polymer informatics by enabling the generation of high-fidelity SEC profiles from fundamental molar mass parameters (M_n_ and M_w_).

Mathematical foundation and distribution models

The SEC simulation framework supports two primary statistical distribution models for molar mass representation:

Log-normal distribution

The log-normal probability density function for molar mass is implemented as:

$$\begin{aligned} f(M; \mu, \sigma) = (1/(M\cdot\sigma\cdot\surd(2\pi))) \cdot exp(-(ln(M) - \mu)^{2}/(2\sigma^{2}))\#\left( S SEQ SuppEq \backslash* MERGEFORMAT 12 \right) \end{aligned}$$

Parameter conversion from target moments (M_n_, M_w_) to distribution parameters (μ, σ) follows:

$$\begin{aligned} \sigma^{2} = ln(Mw/Mn)\#\left( S SEQ SuppEq \backslash* MERGEFORMAT 13 \right) \end{aligned}$$

$$\begin{aligned} \mu= ln(Mn) - \sigma^{2}/2\#\left( S SEQ SuppEq \backslash* MERGEFORMAT 14 \right) \end{aligned}$$

Schulz-Zimm distribution

The Schulz-Zimm (gamma) distribution is parameterized through shape (α) and rate (β) parameters:

$$\begin{aligned} \alpha= 1/(Đ- 1), where Đ= Mw/Mn\#\left( S SEQ SuppEq \backslash* MERGEFORMAT 15 \right) \end{aligned}$$

$$\begin{aligned} \beta= \alpha/Mn\#\left( S SEQ SuppEq \backslash* MERGEFORMAT 16 \right) \end{aligned}$$

Iterative parameter refinement algorithm

The simulation employs a Newton-like optimization procedure to ensure precise reproduction of target molar mass moments:

1. **Initial parameter estimation**: Distribution parameters are calculated from target M_n_ and M_w_ values using theoretical relationships.
2. **Small-scale simulation**: A subset simulation (*n_molecules*/10) generates preliminary molar mass distributions.
3. **Moment calculation**: Simulated M_n_ and M_w_ are computed from the generated distribution.
4. **Error assessment**: Relative errors between simulated and target moments are evaluated.
5. **Parameter adjustment**: Distribution-specific update rules minimize moment discrepancies:
   - Log-normal: μ adjustment for scaling, σ modification for width.
   - Schulz-Zimm: β adjustment for Mn matching, damped α updates for Đ control.
6. **Convergence evaluation**: The process iterates until moment errors fall below specified tolerance (default: 0.5%).

Chromatographic realism enhancement

Volume-mass calibration

Molar mass-to-elution volume conversion employs a linear calibration relationship:

$$\begin{aligned} log₁₀(M) = a\cdot V + b\#\left( S SEQ SuppEq \backslash* MERGEFORMAT 17 \right) \end{aligned}$$

where V represents elution volume, and parameters a (slope) and b (intercept) define the calibration curve. Default values (a = -0.45, b = 10.5) approximate typical SEC column performance (real calibration curves usually follow a higher order but this has no effect on the simulation).

Instrumental effects simulation

The framework incorporates multiple sources of experimental variability:

- **Band broadening**: Gaussian smoothing with adjustable σ parameter simulates column dispersion effects.
- **Peak asymmetry**: Exponential tail addition models non-ideal elution behavior.
- **Baseline drift**: Constant offset addition represents detector baseline variations.
- **Instrumental noise**: Gaussian noise addition simulates detector signal fluctuations.

Histogram construction and processing

Final SEC traces are constructed through:

1. **Binning**: Molar masses are discretized into elution volume bins across the specified range.
2. **Weight-based accumulation**: Histogram construction uses molar masses as bin weights rather than simple counts.
3. **Signal processing**: Sequential application of smoothing, noise addition, and baseline correction.
4. **Data structure**: Output as pandas DataFrame with volume and signal columns compatible with experimental data formats.

Implementation details

Computational optimization

The *make_sec* function implements robust error handling through:

- **Multi-trial strategy**: Up to 50 attempts with incremented random seeds.
- **Adaptive tolerance**: Progressive relaxation of convergence criteria (0.005 → 0.05).
- **Parameter validation**: Input verification and constraint enforcement.

Integration with experimental workflow

The simulation framework seamlessly interfaces with the broader polymer informatics pipeline:

- **Moment preservation**: Generated distributions precisely match experimental *M_n_*/*M_w_* specifications
- **Format compatibility**: Output structures align with experimental SEC data processing workflows
- **Calibration flexibility**: User-definable calibration parameters accommodate diverse column configurations

This SEC simulation capability enables the generation of statistically representative molar mass distributions that maintain experimental fidelity while providing the large-scale datasets necessary for robust machine learning model development.

1. Dataset analysis

PolyMetriX *T_g_*-dataset^[7]^

**
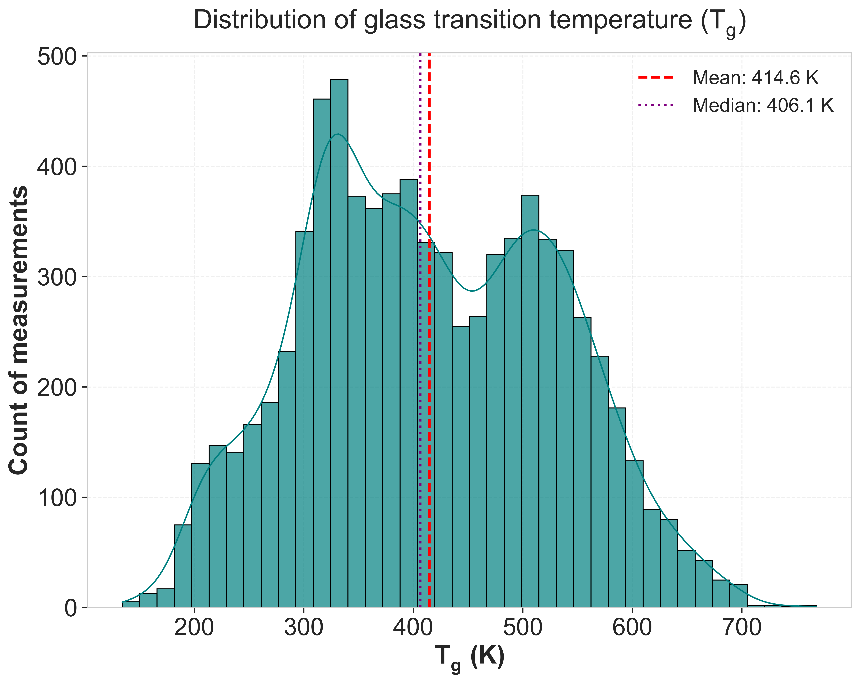
**

**Figure S2**: Plot of the T_g_-value distribution within the curated dataset.


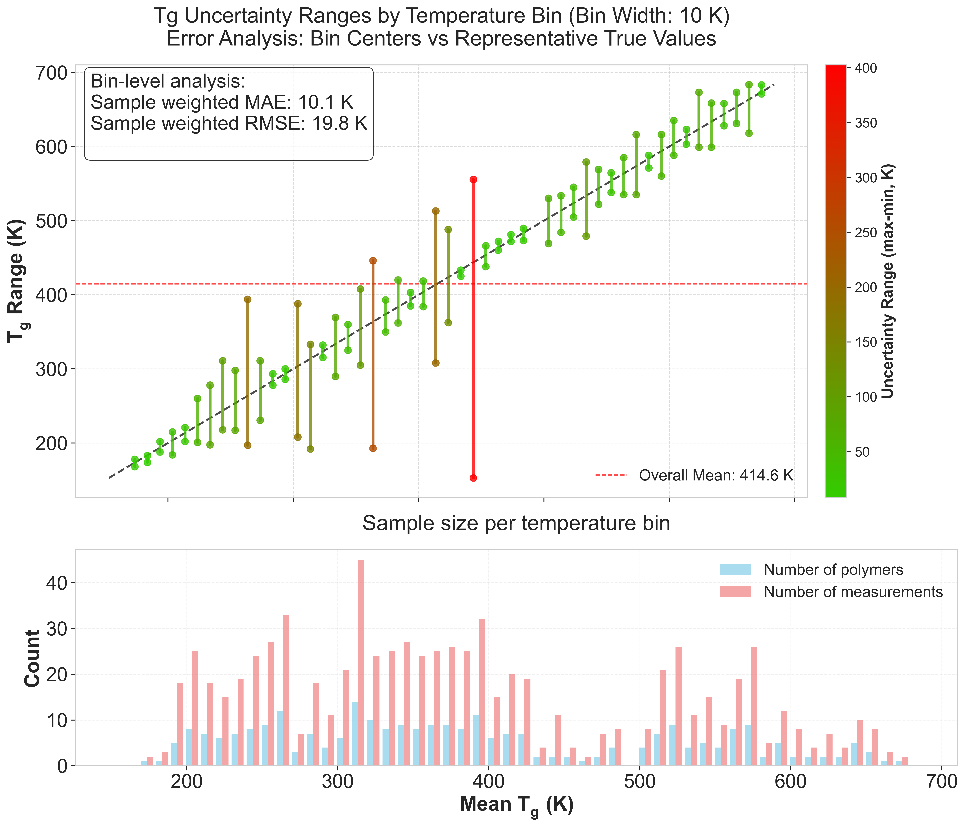


**Figure S3**: Binned range of uncertainties of individual polymers in the dataset, plotted against the mean temperature of the individual polymers. The plotted uncertainty range is calculated by taking the maximum and minimum measurement of all polymers whose mean T_g_-value falls into the respective temperature bin. The calculated MAE is calculated via
$MAE = \frac{\sum_{i}^{n} |Measurement_{i}-Mean_{i}|}{n}$, where Mean_i_ is the mean of all measurements of the respective polymer_i._ The RMSE is calculated respectively.

Baseline model comparison

To validate the efficacy of the hierarchical graph architecture, the proposed model was benchmarked against three standard machine learning approaches. To ensure a fair comparison of chemical feature extraction capabilities, all baseline models utilized 600-dimensional embeddings generated by the pre-trained polyBERT language model as input features.^[8]^

The following baseline models were implemented using the Scikit-learn library^[9]^:

1. Linear regression: A standard Ordinary Least Squares (OLS) regression.
2. Random Forest regressor: An ensemble method configured with 100 estimators.
3. Support Vector Regression (SVR): A kernel-based method utilizing a Radial Basis Function (RBF) kernel (C = 10, ε = 0.1) and standardized input features.

All models were evaluated using the same 5-fold cross-validation splits as the primary GCN model on the large-scale *T*_g_-dataset. The results, summarized in **Table S1**, demonstrate that while the baseline models achieve reasonable correlation (R² ≈ 0.83), the structure-aware GCN significantly outperforms them, reducing the mean absolute error by approximately 7 K (>20% improvement).

**Table S1:** Performance comparison between polyBERT-based baseline models and the proposed structure-aware GCN.

| Model architecture | Input representation | R² score | MAE (K) |
| --- | --- | --- | --- |
| Linear regression | polyBERT embedding | 0.835 ± 0.007 | 33.64 |
| Random Forest | polyBERT embedding | 0.832 ± 0.010 | 33.58 |
| SVR (RBF Kernel) | polyBERT embedding | 0.828 ± 0.004 | 33.32 |
| Structure-aware GCN (current study) | **Hierarchical graph ensemble** | **0.89 ± 0.01** | **26.24** |

Curated dataset by the authors

**
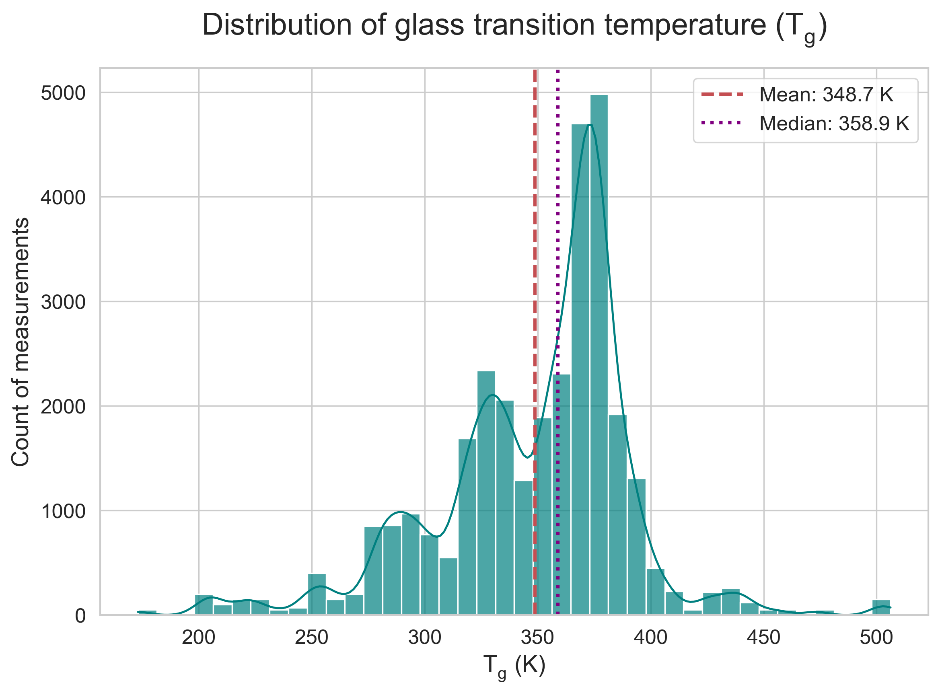
**

**Figure S4**: Plot of the T_g_-distribution within the curated dataset.


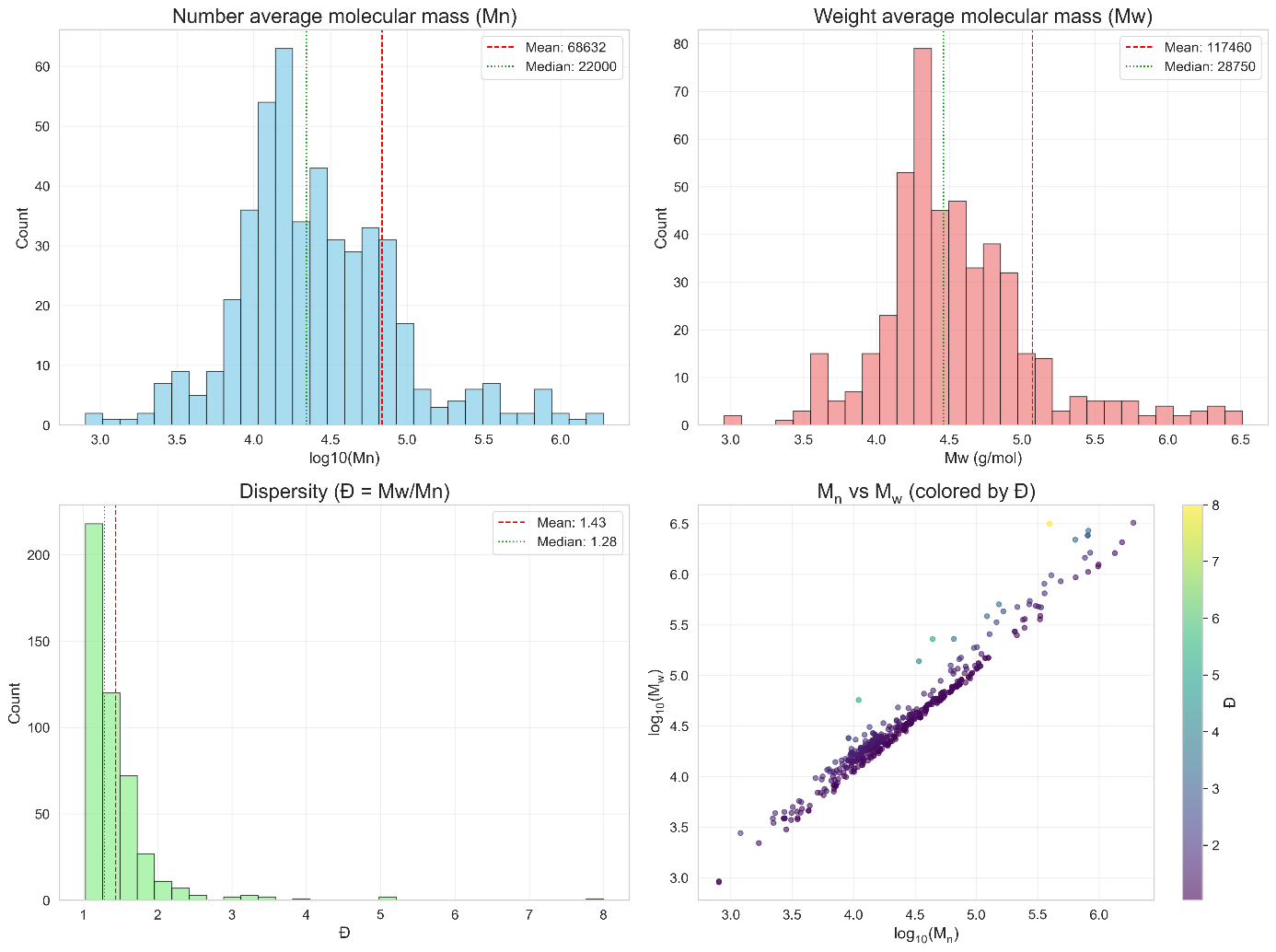


**Figure S5**: Plot of the distribution and relationships of M_n_ and M_w_ within the dataset.


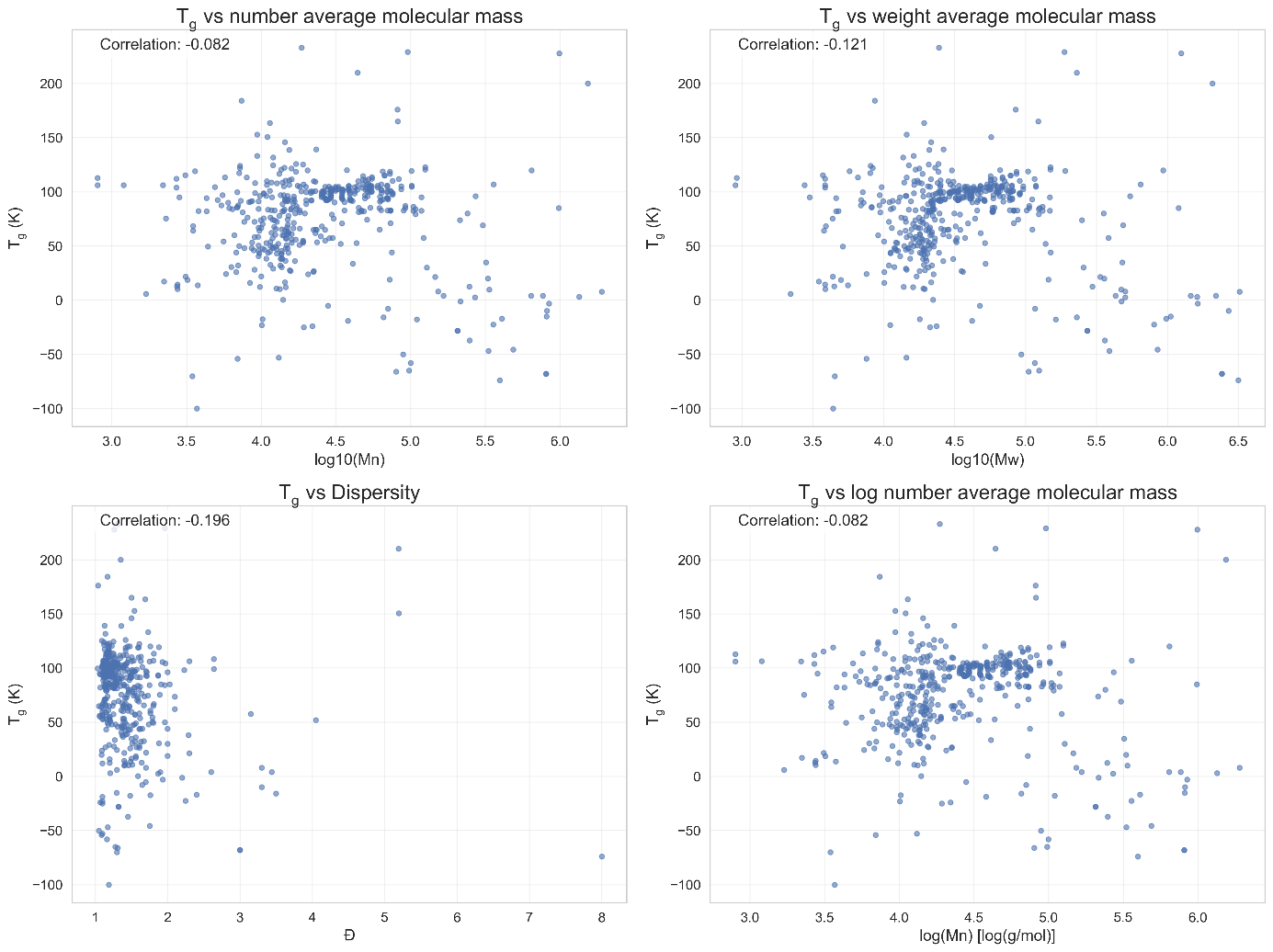


**Figure S6**: Plotting the T_g_-values of the polymers in the dataset against the molar mass parameters M_n_ or M_w_ shows no direct correlation, which is important to prevent a induced bias in the training data.


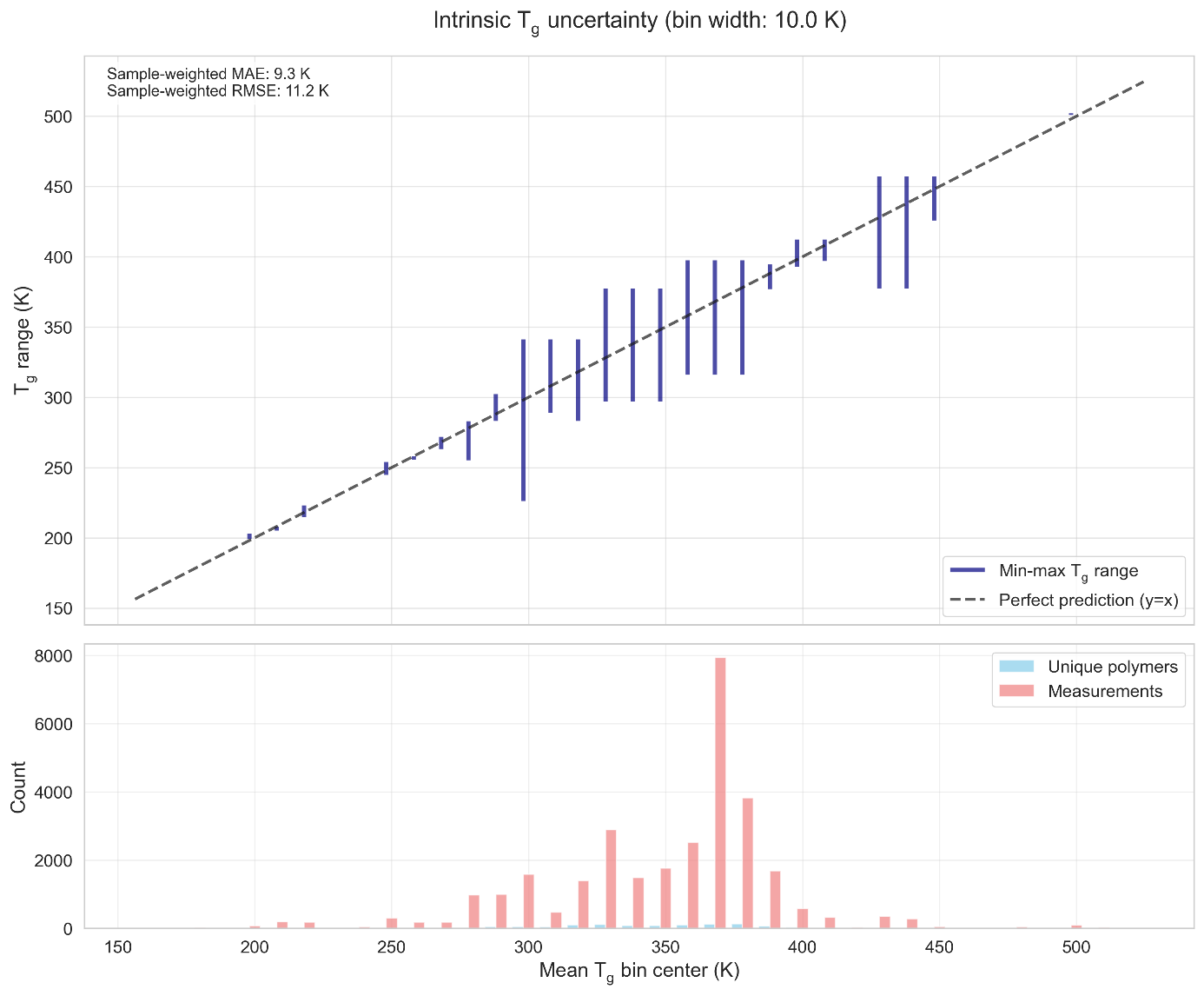


**Figure S7**: Binned range of uncertainties of individual polymers in the dataset, plotted against the mean temperature of the individual polymers.

1. Visualization of graphs of the synthetic polymer architecture dataset

The following graph visualization represents randomly sampled graphs of the synthetic polymer dataset. The synthetic polymer graphs for the structure awareness task do not require labeled monomers, as this could lead to a wrong interpretation of the network (*e.g.* the presence of a specific monomer is linked to specific architecture). To prevent this the monomer features per graph are randomly generated, to force the network to interfere the classes from the graph topology. In the following representations the different repeating units are only differentiated per graph and are labeled A, B, C, D, … but each label can be a different type (feature vector) in each graph.

Linear homopolymers


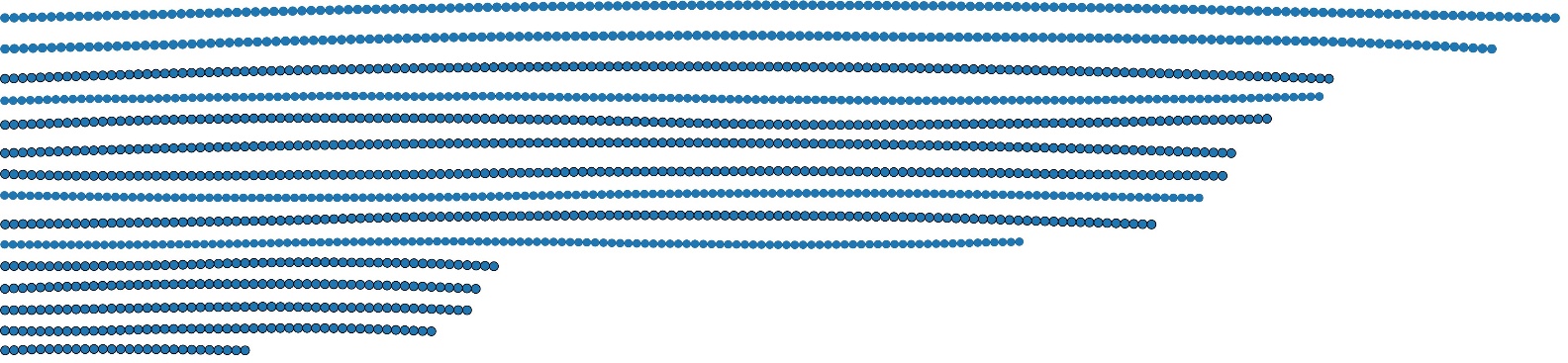

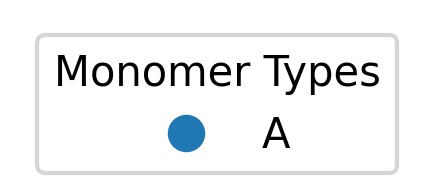


Linear random copolymers


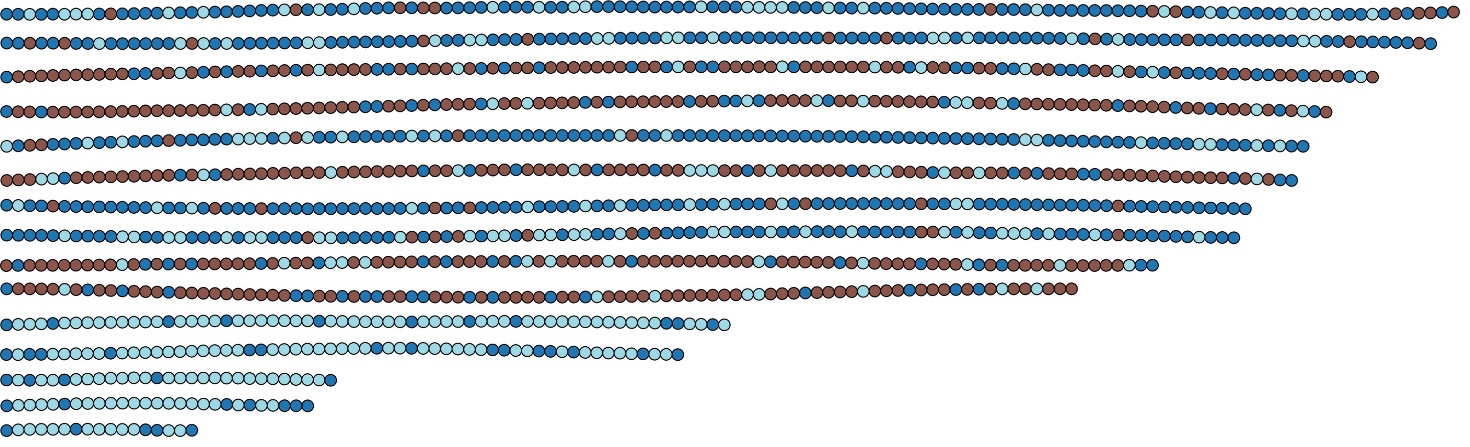

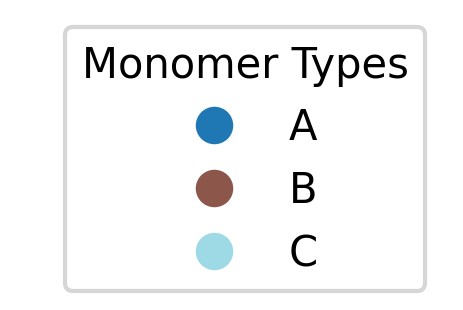


Linear gradient copolymers


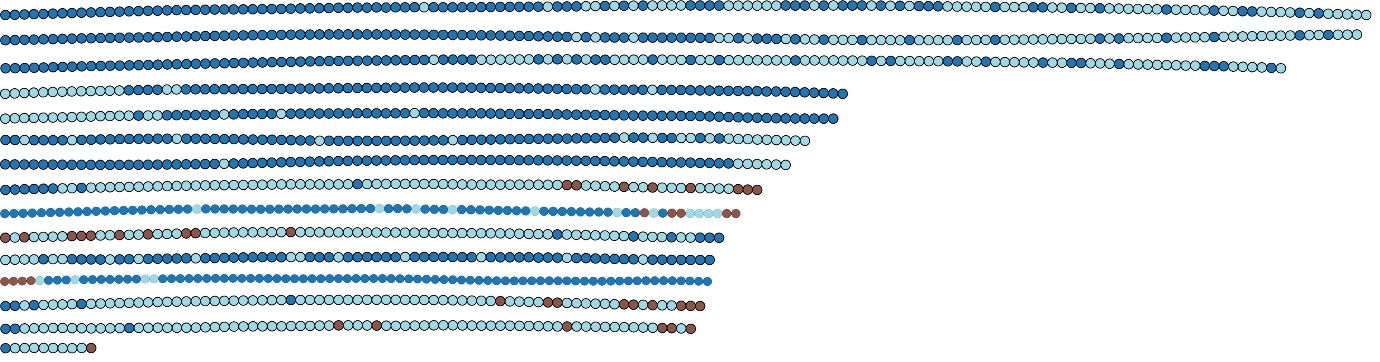

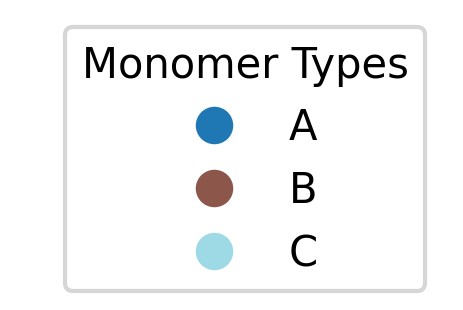


Linear block copolymers


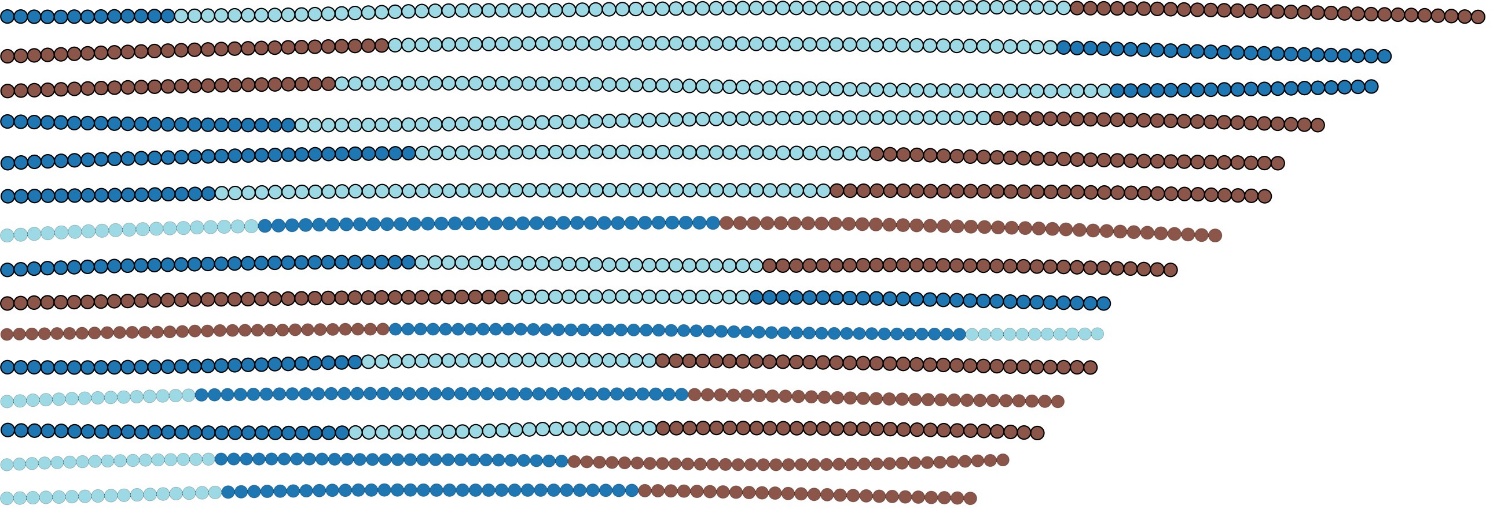

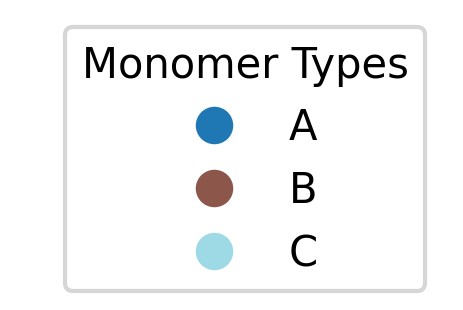


Branching homopolymers


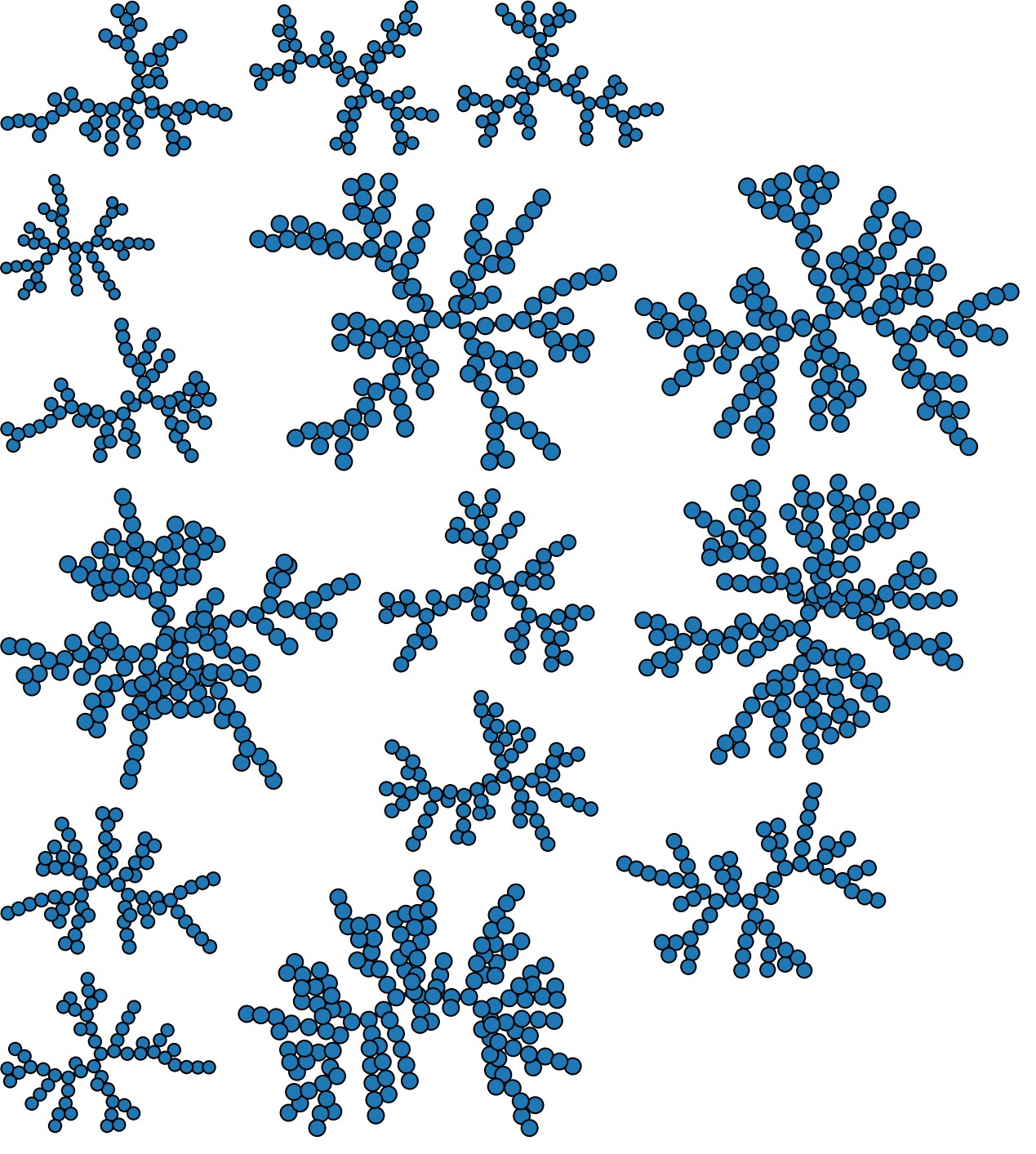

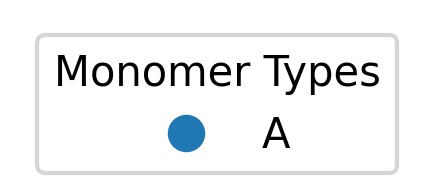


Branching random copolymers


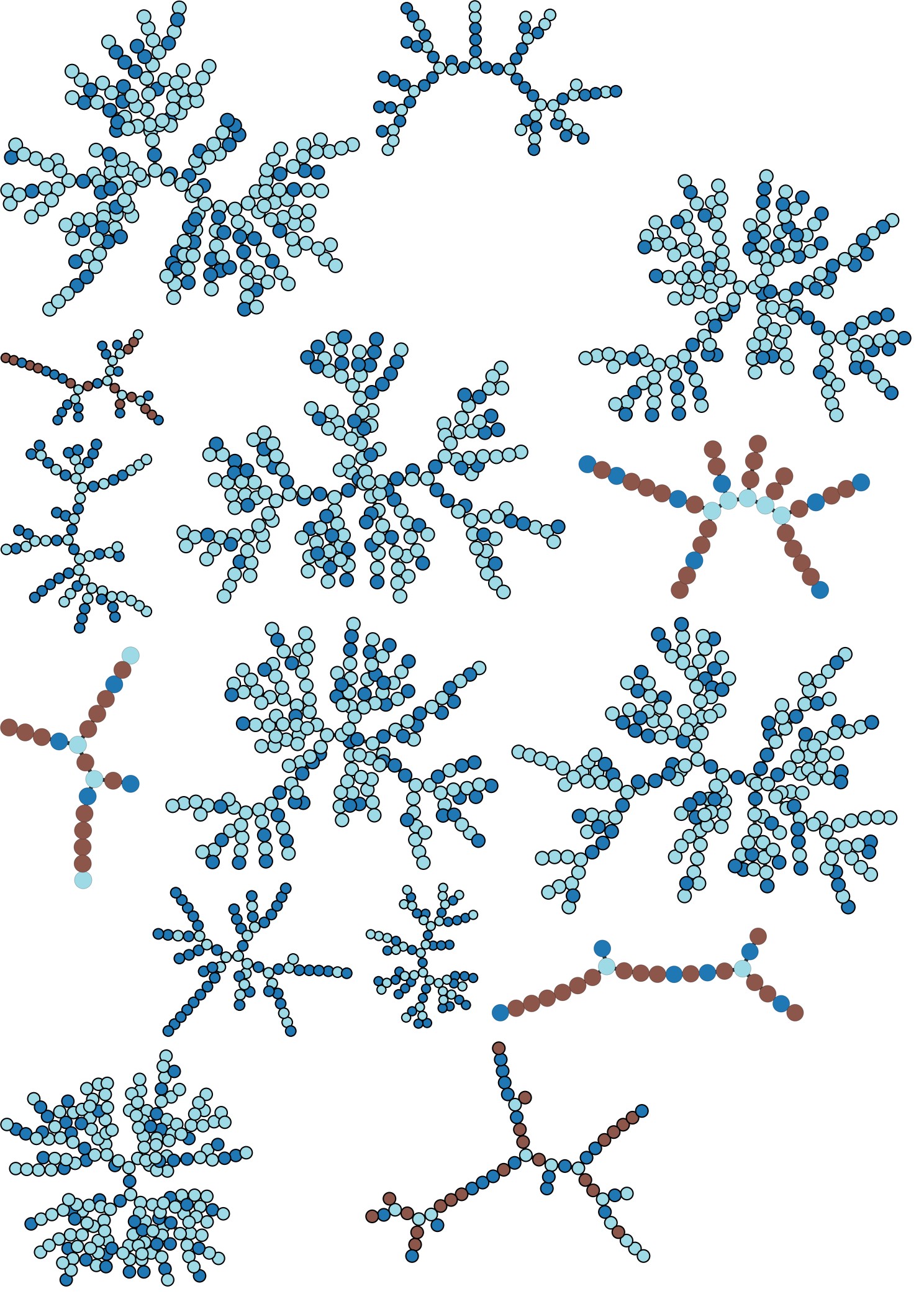

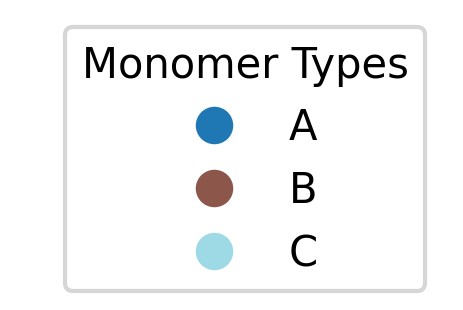


Branching gradient copolymers


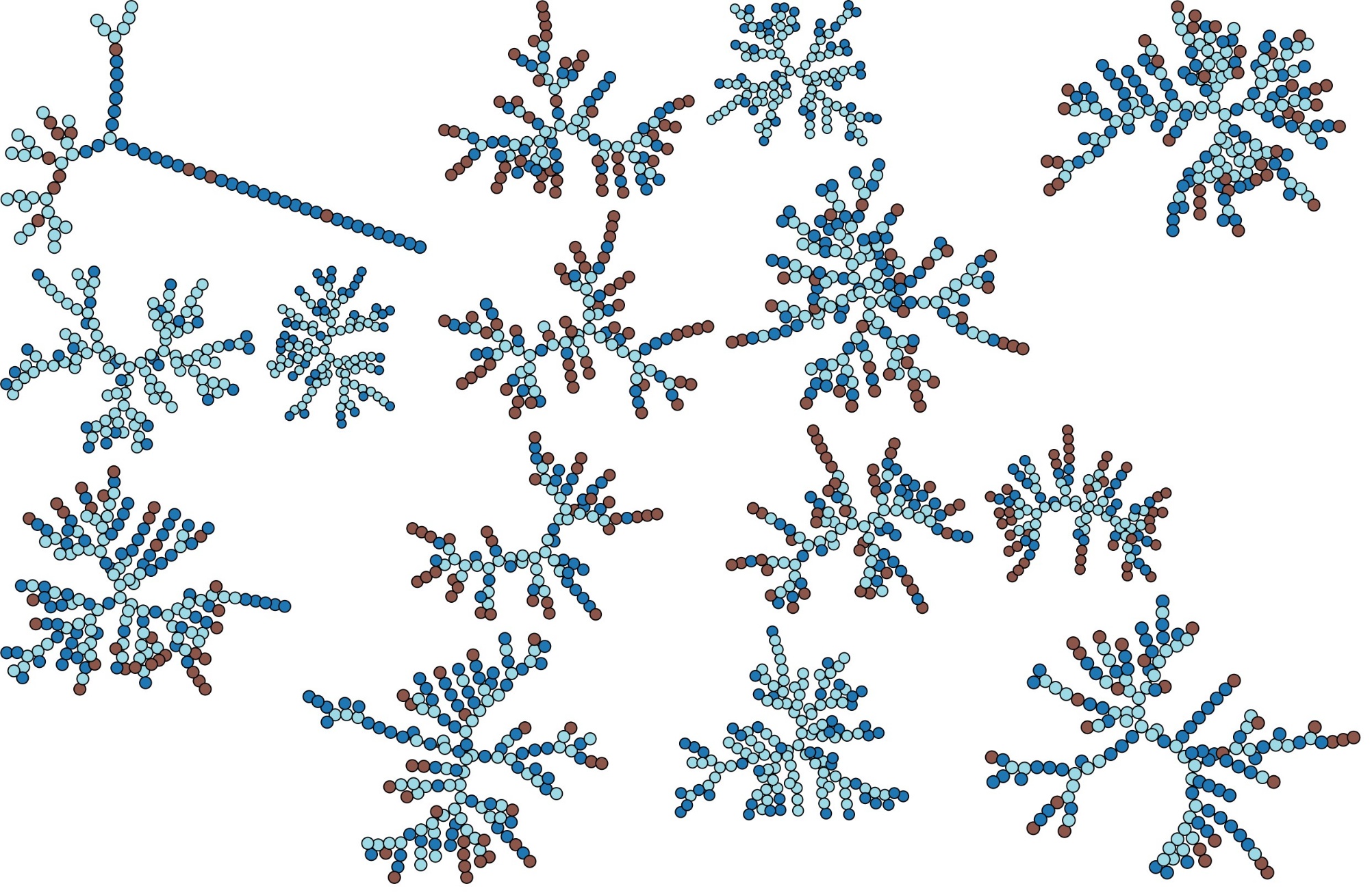

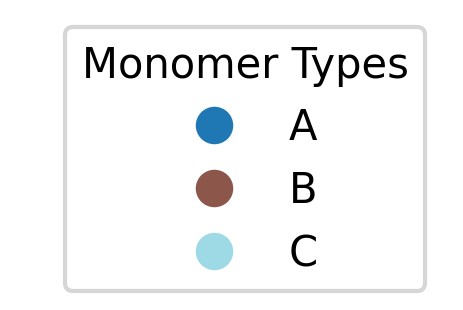


Branching block copolymers


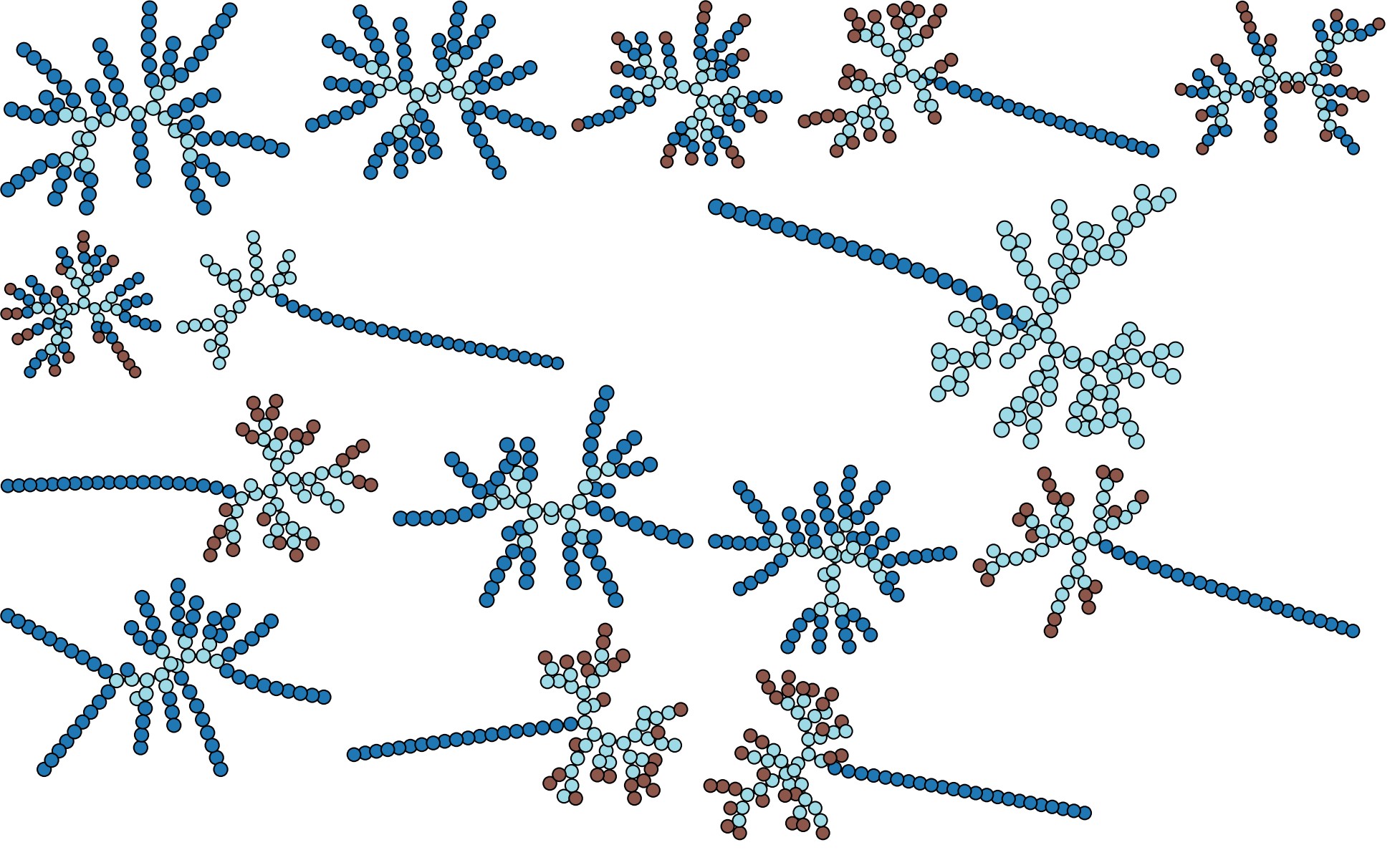

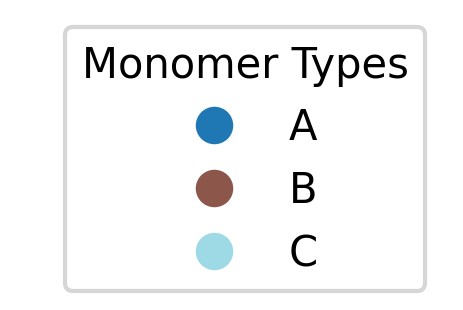


Cross-linked homopolymers


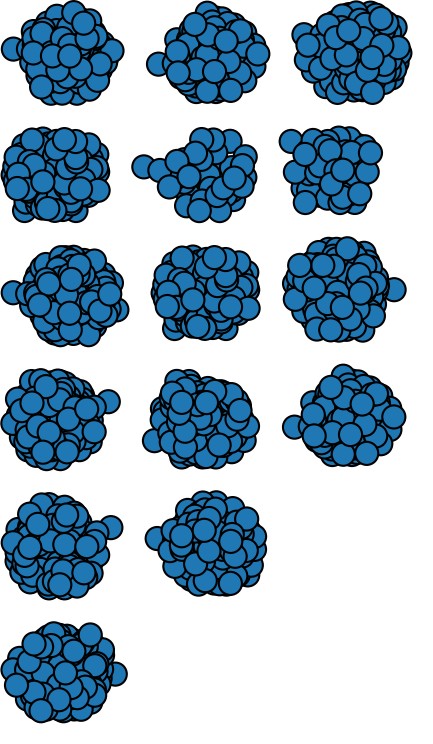

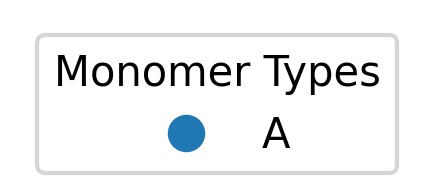


Cross-linked random copolymers


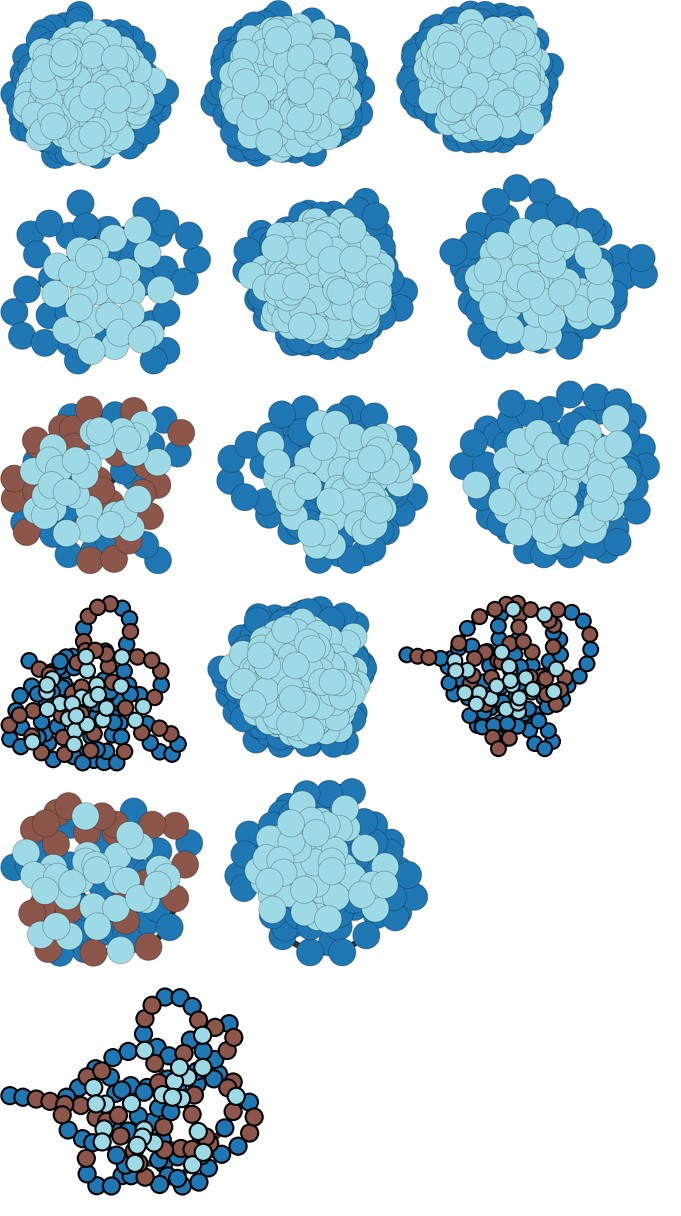

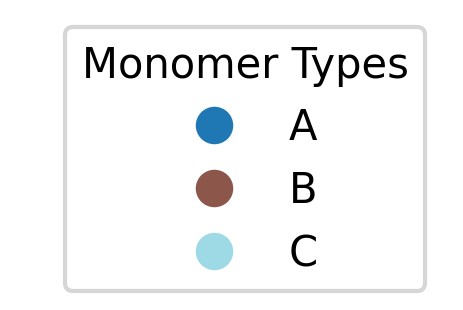


Cross-linked gradient copolymers


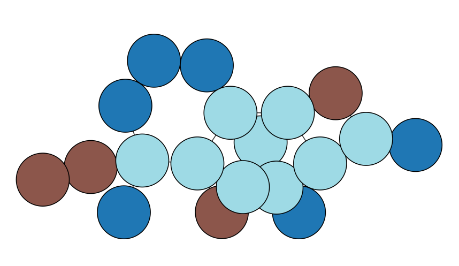

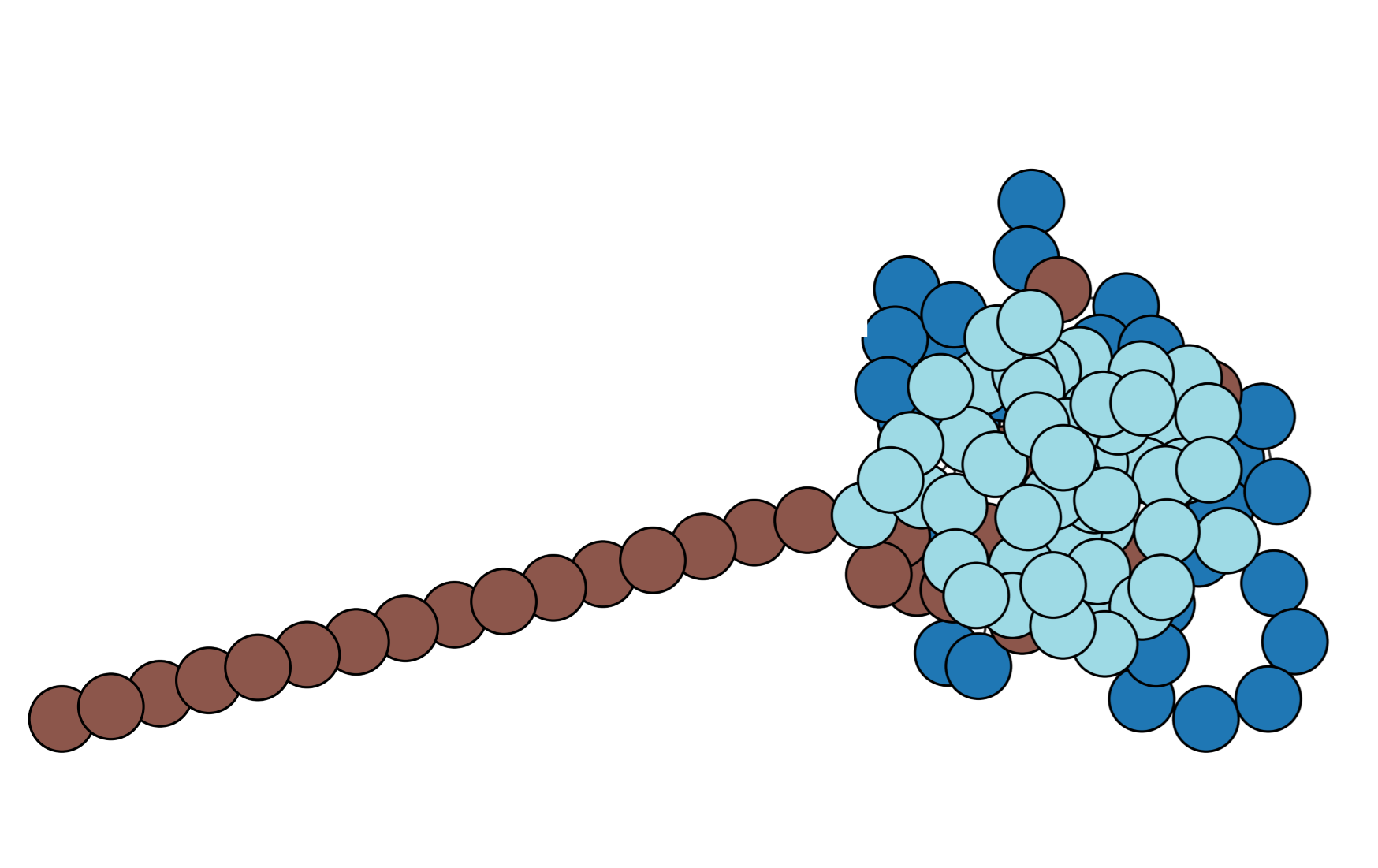

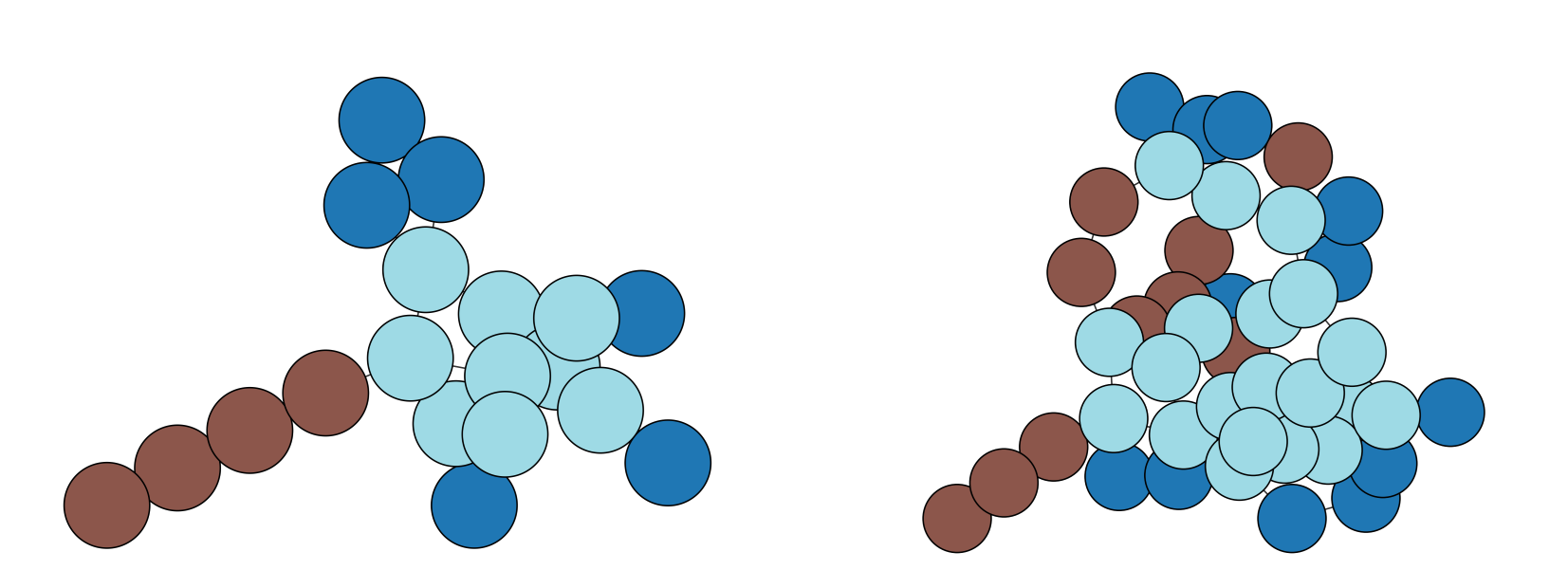

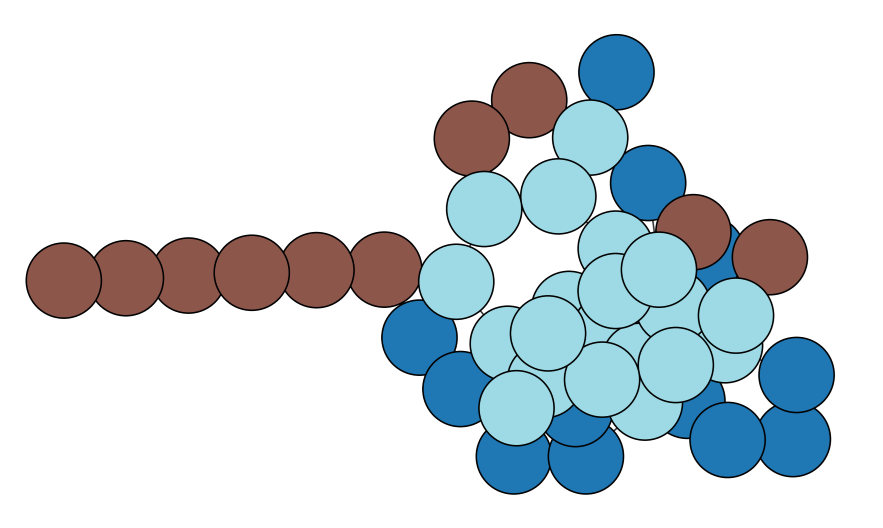

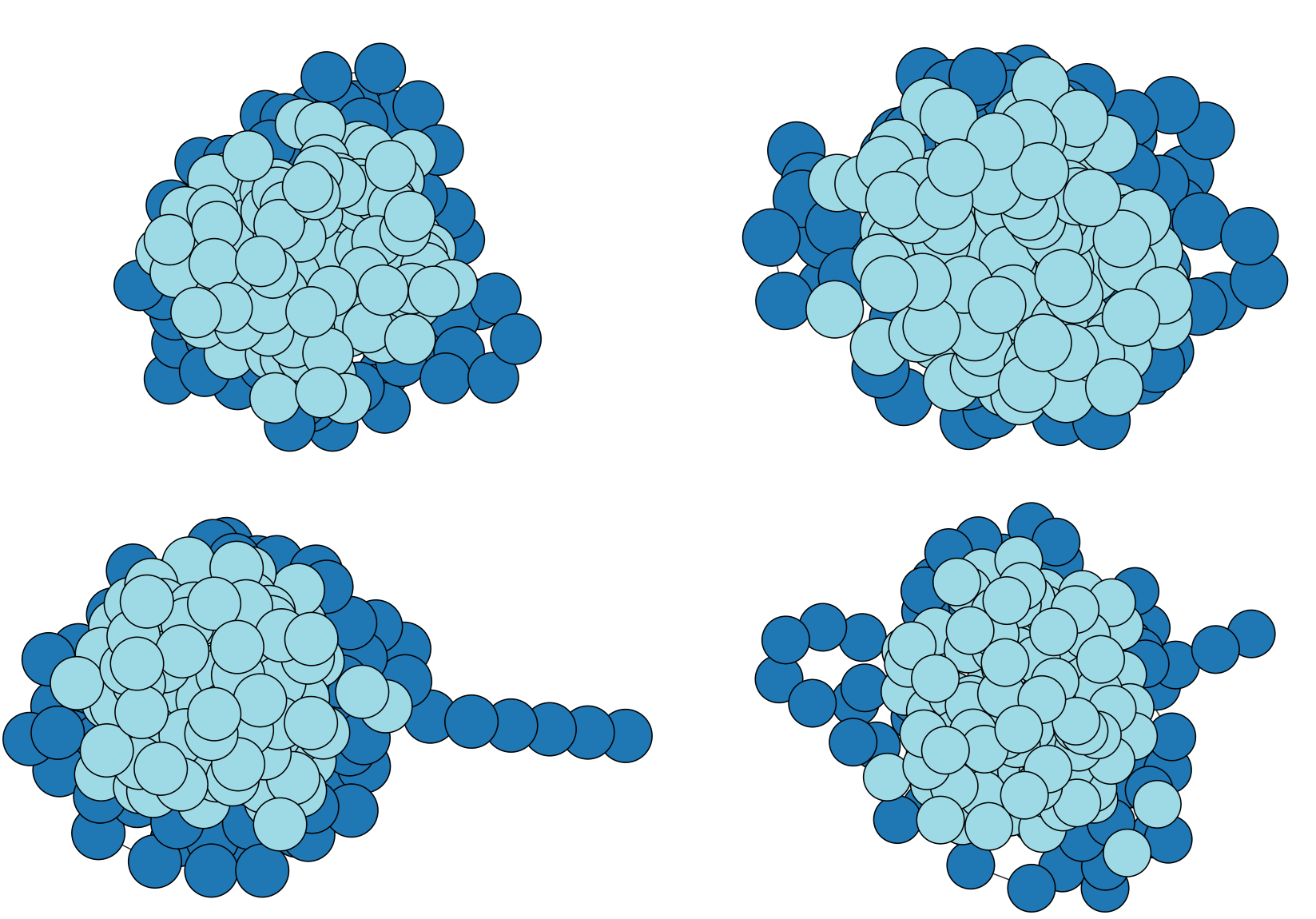

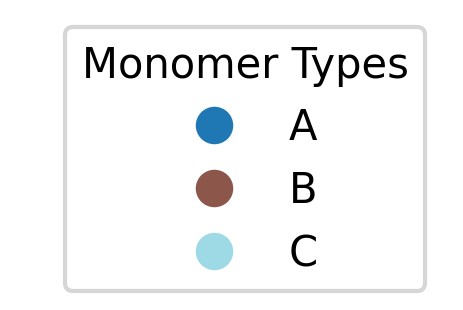


Star homopolymers


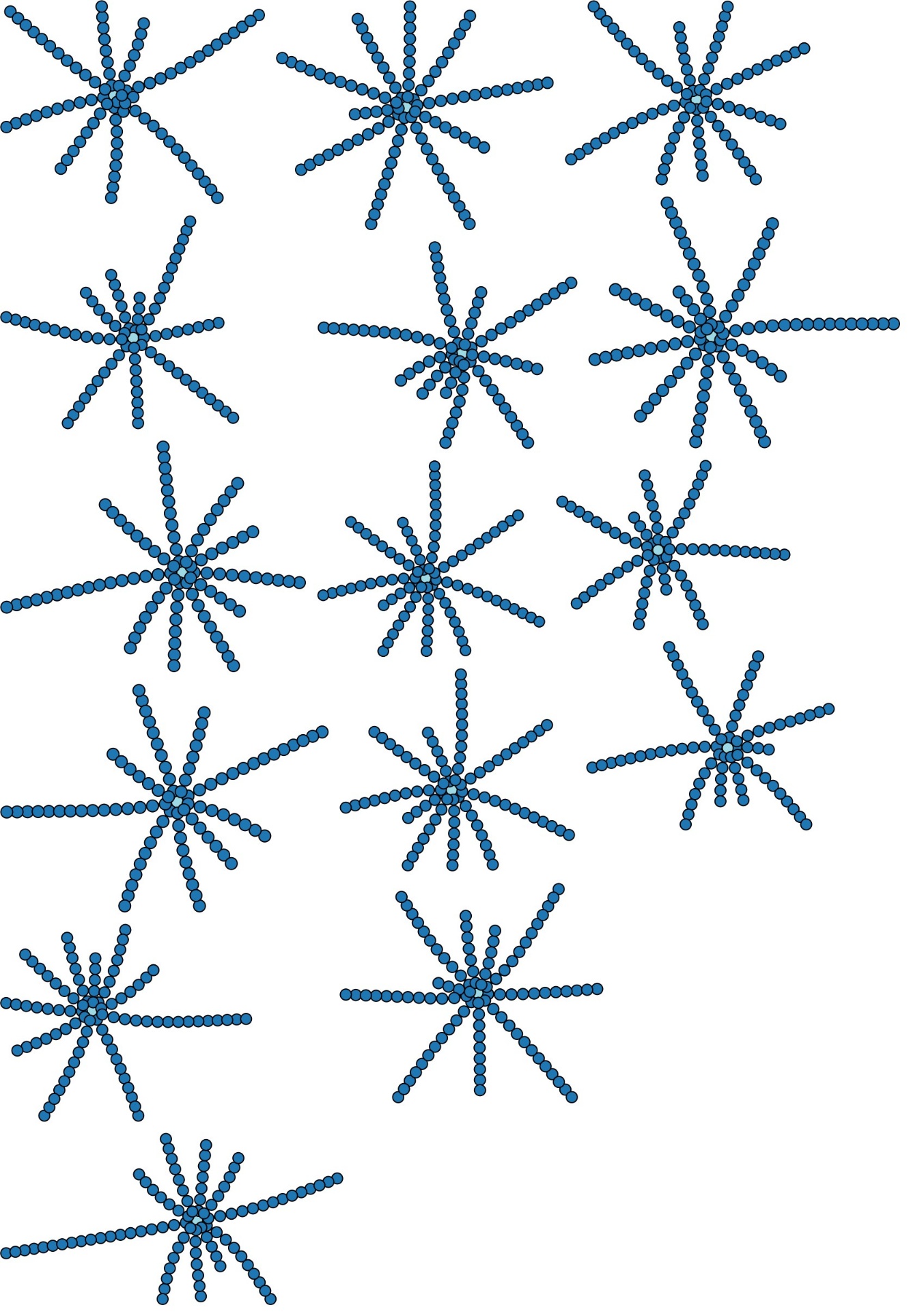

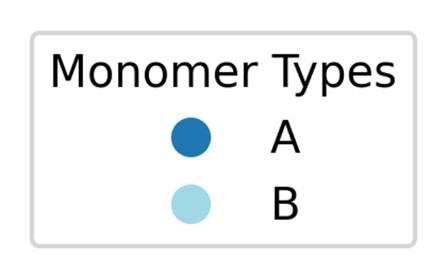


Star random copolymers


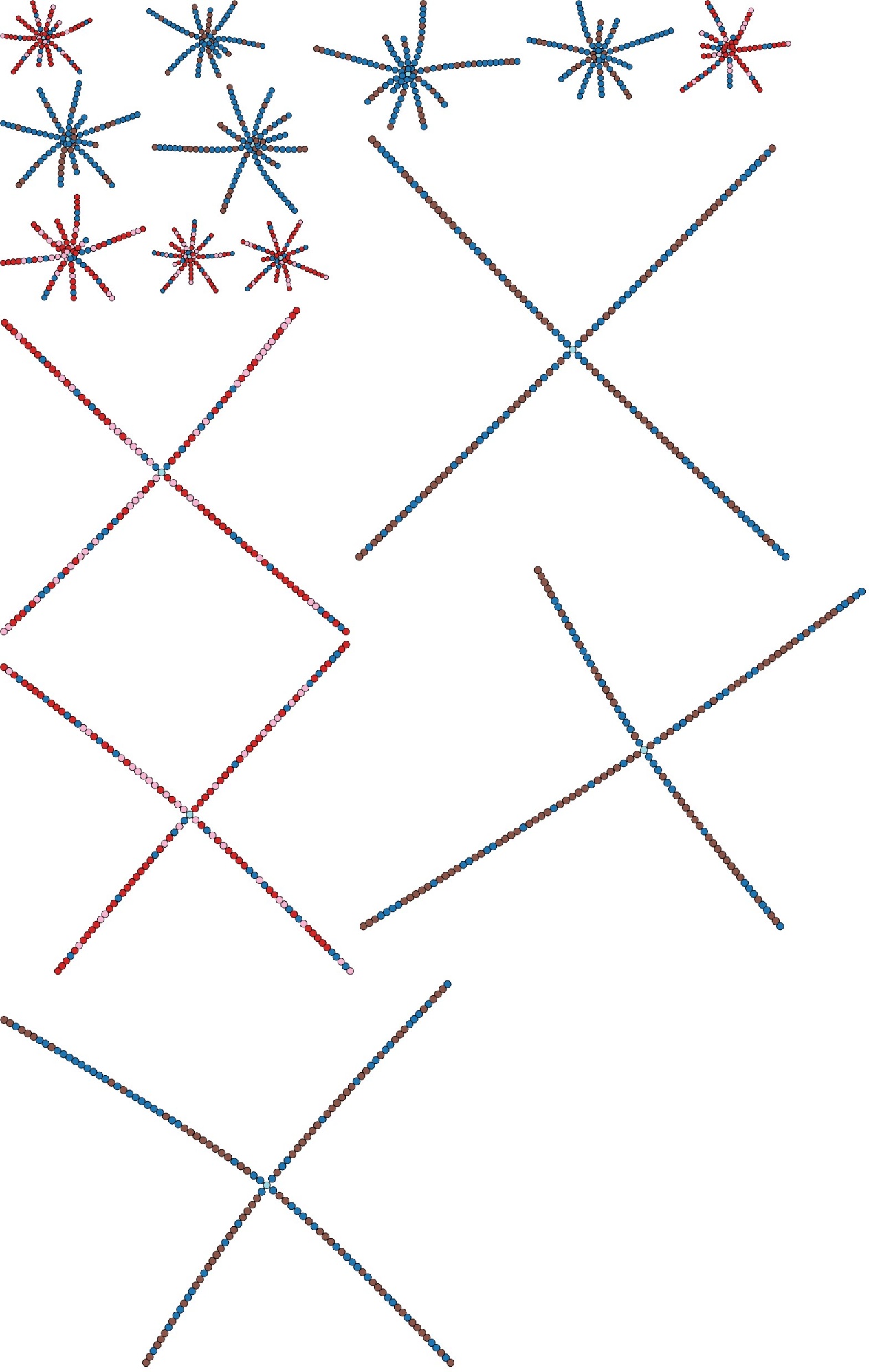

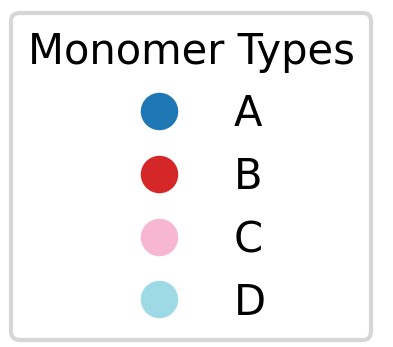


Star gradient copolymers


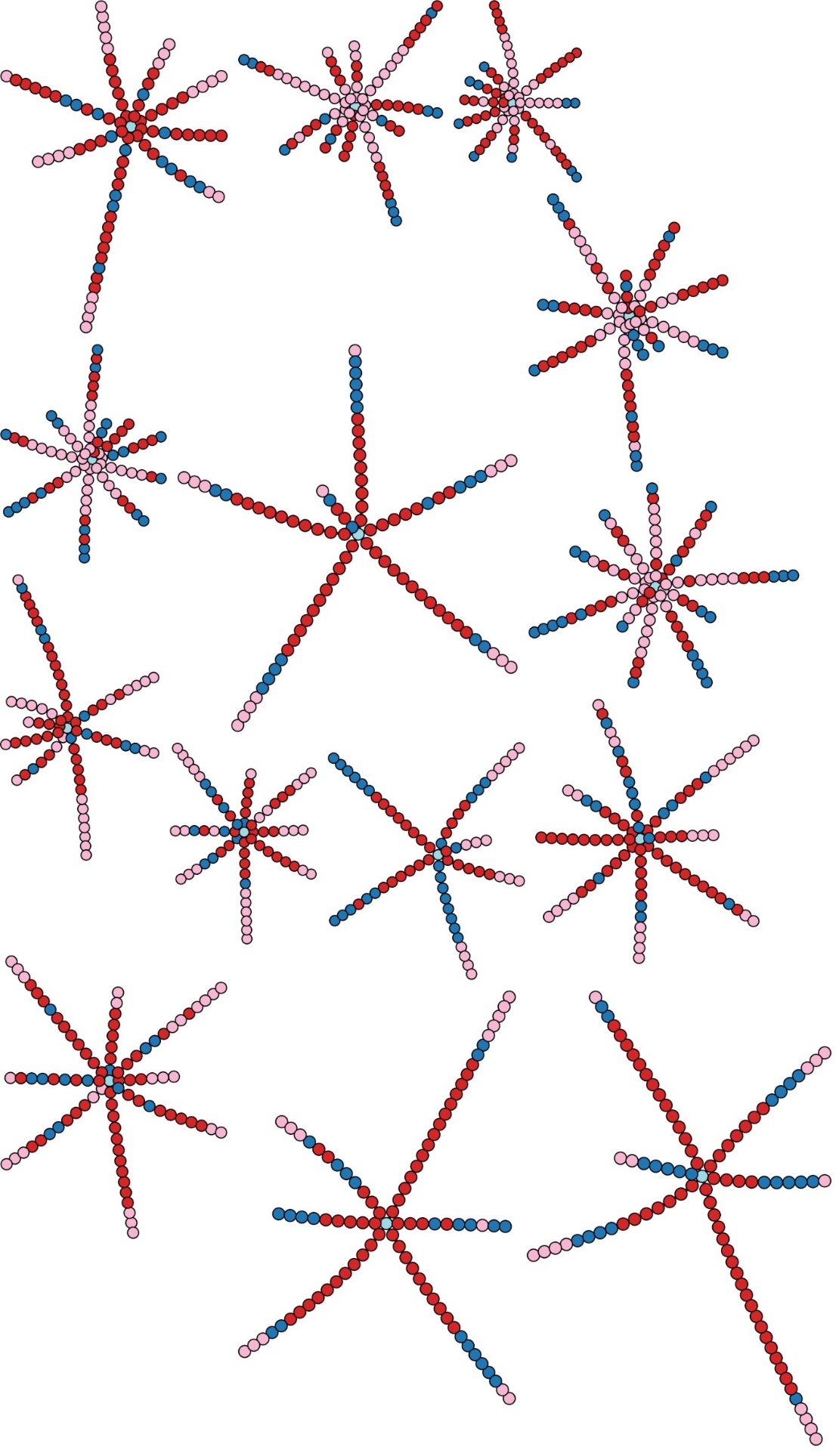

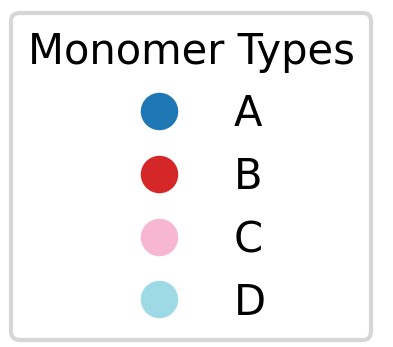


Star block copolymers


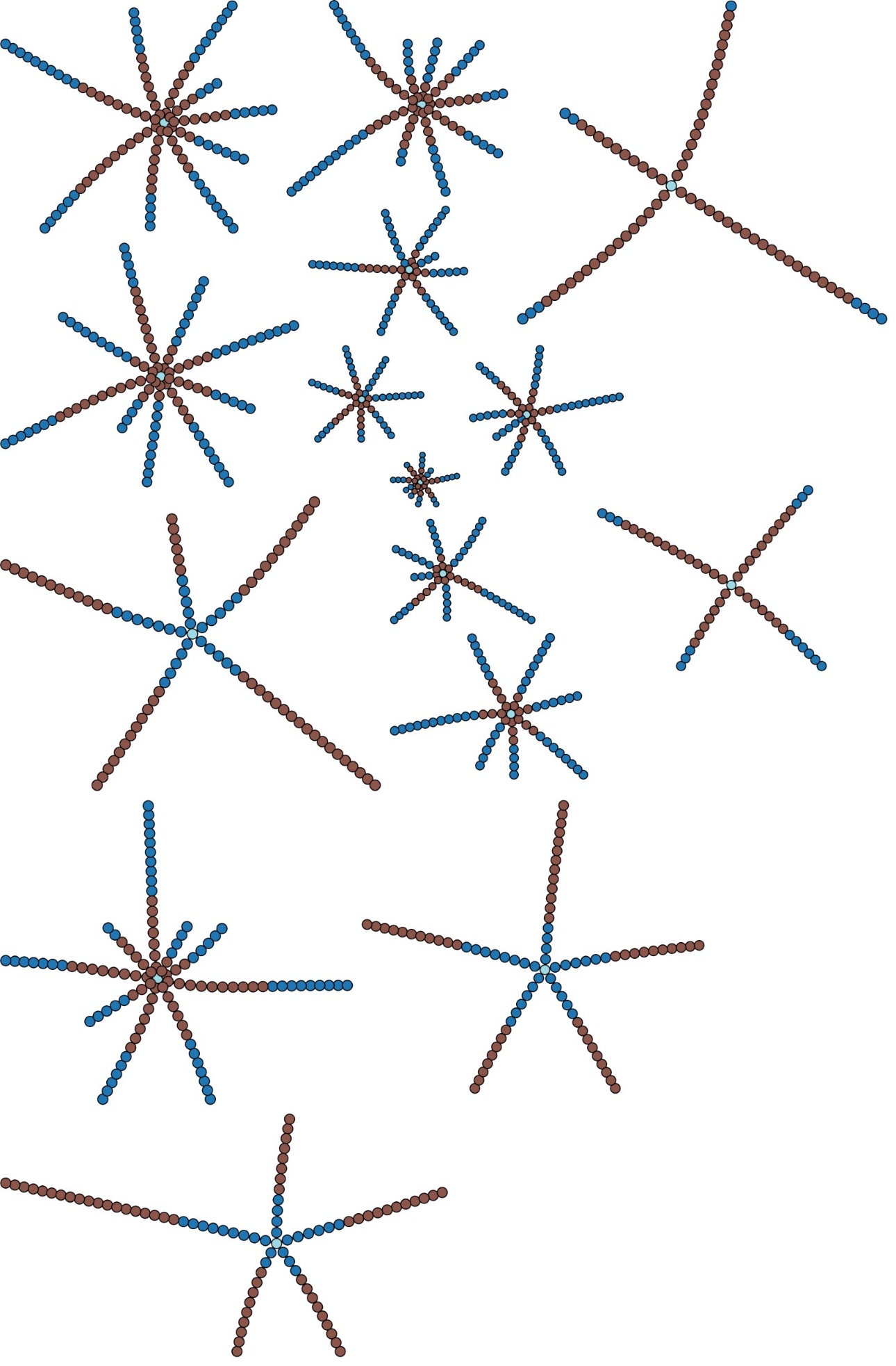

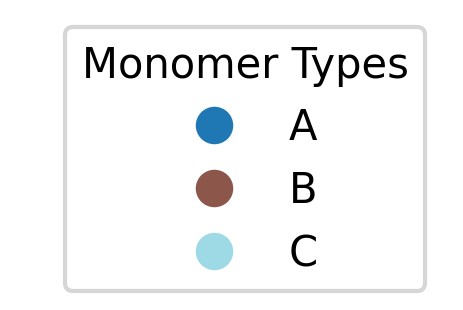


1. Usage of generative AI during the manuscript preparation:

For full transparency we declare that generative AI, namely ChatGPT-o3 was utilized for language correction using this prompt predececing individual text parts:

*“You are a proofreader with a professional background in scientific writing. Your goal is to check text passages of scientific publications for consistent and correct language. The scientific writing style should be precise, technical, and authoritative while maintaining clarity and accessibility. It should present complex technical information in a structured, logical manner with a balanced tone. It should have clear, concise language that explains sophisticated concepts without unnecessary jargon.

You will respond with a revised version.

You will NEVER add or remove any content you will focus ONLY on the language.
You will NEVER perform calculations yourself, instead use placeholders (<calculate>)*

*Examples:

Input:
In our experiment we added compound X32 to Water. Then we added 32 g of table salt. Then we heated the solution with a reflux condenser to boiling point until everything is clear. We then waited until its cold and then filtrated the precipitation. As a result we got 10g of the wanted produkt

Expected response:
In our experiment, we added compound X32 (<quantity>) to water (<quantity>), followed by the addition of sodium chloride (32 g, <mols>). The solution was then refluxed until all solids completely dissolved. It was subsequently cooled to room temperature, after which the precipitate was filtered. 10 g of the desired product was obtained.

Input:
The laser beam was focused on the metal surface for 5 seconds. Then we measured the temperature rise using a thermal camera. After that the sample was cooled down naturally and the structural changes were analyzed with an electron microscope. The images showed small cracks near the irradiated spot.*

*Expected response:
The laser beam was focused on the metal surface for a duration of 5 seconds. The temperature rise was subsequently measured using a thermal camera. Following natural cooling of the sample, structural changes were examined with an electron microscope. Images revealed micro-cracks in the vicinity of the irradiated area.

The text to correct is:*

*<text passage>”*

We declare that the generated text was never used as created but used to identify sections within our text, that we have to clarify or where simple grammar or spelling mistakes were made.

References

[1] M. Fey, J. E. Lenssen, *Fast Graph Representation Learning with PyTorch Geometric.* http://arxiv.org/pdf/1903.02428.

[2] W. L. Hamilton, R. Ying, J. Leskovec, *Inductive Representation Learning on Large Graphs.* **07/06/2017**.

[3] T. Akiba, S. Sano, T. Yanase, T. Ohta, M. Koyama, “Optuna”, in *Proceedings of the 25th ACM SIGKDD International Conference on Knowledge Discovery & Data Mining.* ACM, New York, NY, USA. **07252019**, p. 2623 ff.

[4] D. T. Gillespie, *J. Comput. Phys.* **1976**, *22*, 403.

[5] S. K. Lam, A. Pitrou, S. Seibert, “Numba”, in *Proceedings of the Second Workshop on the LLVM Compiler Infrastructure in HPC.* ACM, New York, NY, USA. **11152015**, p. 1 ff.

[6] A. A. Hagberg, D. A. Schult, P. J. Swart, “Exploring Network Structure, Dynamics, and Function using NetworkX”, in *Proceedings of the 7th Python in Science Conference.* SciPy. **2008**, p. 11 ff.

[7] S. Kunchapu, K. M. Jablonka, *PolyMetriX: An Ecosystem for Digital Polymer Chemistry.* **2025.** 10.26434/chemrxiv-2025-s2f2r.

[8] C. Kuenneth, R. Ramprasad, *Nat. Commun.* **2023**, *14*, 4099.

[9] F. Pedregosa, G. Varoquaux, A. Gramfort, V. Michel, B. Thirion, O. Grisel, M. Blondel, P. Prettenhofer, R. Weiss, V. Dubourg, J. Vanderplas, A. Passos, D. Cournapeau, M. Brucher, M. Perrot, E. Duchesnay, *Journal of Machine Learning Research.* **2011**, *12*, 2825.
